# The potential health and economic value of SARS-CoV-2 vaccination alongside physical distancing in the UK: a transmission model-based future scenario analysis and economic evaluation

**DOI:** 10.1016/S1473-3099(21)00079-7

**Published:** 2021-07

**Authors:** Frank G Sandmann, Nicholas G Davies, Anna Vassall, W John Edmunds, Mark Jit, Centre for the Mathematical Modelling of Infectious Diseases COVID-19 working group, Fiona Yueqian Sun, C Julian Villabona-Arenas, Emily S Nightingale, Alicia Showering, Gwenan M Knight, Katharine Sherratt, Yang Liu, Kaja Abbas, Sebastian Funk, Akira Endo, Joel Hellewell, Alicia Rosello, Rachel Lowe, Matthew Quaife, Amy Gimma, Oliver Brady, Jack Williams, Simon R Procter, Rosalind M Eggo, Yung-Wai Desmond Chan, James D Munday, Rosanna C Barnard, Georgia R Gore-Langton, Nikos I Bosse, Naomi R Waterlow, Charlie Diamond, Timothy W Russell, Graham Medley, Stefan Flasche, Katherine E Atkins, Kiesha Prem, David Simons, Megan Auzenbergs, Damien C Tully, Christopher I Jarvis, Kevin van Zandvoort, Sam Abbott, Carl A B Pearson, Thibaut Jombart, Sophie R Meakin, Anna M Foss, Adam J Kucharski, Billy J Quilty, Hamish P Gibbs, Samuel Clifford, Petra Klepac

**Affiliations:** a Centre for Mathematical Modelling of Infectious Diseases, London School of Hygiene & Tropical Medicine, London, UK; b Statistics, Modelling and Economics Department, National Infection Service, Public Health England, London, UK; c School of Public Health, University of Hong Kong, Hong Kong Special Administrative Region, China

## Abstract

**Background:**

In response to the COVID-19 pandemic, the UK first adopted physical distancing measures in March, 2020. Vaccines against SARS-CoV-2 became available in December, 2020. We explored the health and economic value of introducing SARS-CoV-2 immunisation alongside physical distancing in the UK to gain insights about possible future scenarios in a post-vaccination era.

**Methods:**

We used an age-structured dynamic transmission and economic model to explore different scenarios of UK mass immunisation programmes over 10 years. We compared vaccinating 75% of individuals aged 15 years or older (and annually revaccinating 50% of individuals aged 15–64 years and 75% of individuals aged 65 years or older) to no vaccination. We assumed either 50% vaccine efficacy against disease and 45-week protection (worst-case scenario) or 95% vaccine efficacy against infection and 3-year protection (best-case scenario). Natural immunity was assumed to wane within 45 weeks. We also explored the additional impact of physical distancing on vaccination by assuming either an initial lockdown followed by voluntary physical distancing, or an initial lockdown followed by increased physical distancing mandated above a certain threshold of incident daily infections. We considered benefits in terms of quality-adjusted life-years (QALYs) and costs, both to the health-care payer and the national economy. We discounted future costs and QALYs at 3·5% annually and assumed a monetary value per QALY of £20 000 and a conservative long-run cost per vaccine dose of £15. We explored and varied these parameters in sensitivity analyses. We expressed the health and economic benefits of each scenario with the net monetary value: QALYs × (monetary value per QALY) – costs.

**Findings:**

Without the initial lockdown, vaccination, and increased physical distancing, we estimated 148·0 million (95% uncertainty interval 48·5–198·8) COVID-19 cases and 3·1 million (0·84–4·5) deaths would occur in the UK over 10 years. In the best-case scenario, vaccination minimises community transmission without future periods of increased physical distancing, whereas SARS-CoV-2 becomes endemic with biannual epidemics in the worst-case scenario. Ongoing transmission is also expected in intermediate scenarios with vaccine efficacy similar to published clinical trial data. From a health-care perspective, introducing vaccination leads to incremental net monetary values ranging from £12·0 billion to £334·7 billion in the best-case scenario and from –£1·1 billion to £56·9 billion in the worst-case scenario. Incremental net monetary values of increased physical distancing might be negative from a societal perspective if national economy losses are persistent and large.

**Interpretation:**

Our model findings highlight the substantial health and economic value of introducing SARS-CoV-2 vaccination. Smaller outbreaks could continue even with vaccines, but population-wide implementation of increased physical distancing might no longer be justifiable. Our study provides early insights about possible future post-vaccination scenarios from an economic and epidemiological perspective.

**Funding:**

National Institute for Health Research, European Commission, Bill & Melinda Gates Foundation.

## Introduction

In early 2020, physical distancing (or social distancing) measures were adopted in at least 149 countries in response to the widespread community transmission of SARS-CoV-2.^1^, ^2^ Physical distancing measures included closures of schools, non-essential retail, hospitality venues, and workplaces; orders to stay at home and keep a distance of 1–2 m from individuals from different households; restrictions on mass gatherings and movement (including public transport); and isolation of symptomatic cases and quarantining of exposed individuals.^2^ The UK Government first adopted similar measures in March, 2020, following a rapid rise in cases of COVID-19 and the prospect of the health-care system becoming overwhelmed.^3^

The mandatory measures of physical distancing were implemented in the early stages of the pandemic on the basis of the high transmissibility of SARS-CoV-2 and burden of COVID-19, uncertainties about COVID-19 epidemiology,^1^ the absence of effective pharmaceutical interventions against COVID-19, and the imperative to save lives under the rule of rescue.^4^ Nonetheless, the pandemic response has led to large contractions of the global economy, and the largest contraction in the UK economy since monthly records began in 1997, with gross domestic product (GDP) falling by 5·8% in March, 2020, and by 20·4% in April, 2020,^5^ and being forecast to fall by 11% in 2020–21.^6^ So far, COVID-19 and associated policy responses have cost the UK's economy at least £280 billion in 2020–21, which is nearly 14% of the annual GDP,^6^ without including the full macro-economic impact of both response and disease.

**Research in context**

**Evidence before this study**

We searched PubMed and *medRxiv* for economic evaluations of SARS-CoV-2 vaccines with the search string “(coronavirus OR COVID OR SARS-CoV-2) AND (vaccin* OR immunisation) AND ((economic evaluation) OR (cost effectiveness analysis)) AND 2020[dp]” on Sept 21, 2020, and last on Nov 29, 2020, with no language restrictions. We found one preprint publication that valued health outcomes in monetary terms and explored the additional impact of vaccines in a cost–benefit analysis of physical distancing for the USA, and a preprint that used a static Markov model to explore the cost-effectiveness of vaccines in the USA. No study focused on vaccines in a full economic evaluation using a dynamic modelling framework.

**Added value of this study**

With several vaccines being authorised for supply in the UK, our study is, to our knowledge, one of the first to explore the health and economic value of introducing a national SARS-CoV-2 immunisation programme. A programme with high vaccine effectiveness and long-lasting protection might minimise community transmission without further need of increased physical distancing, but a vaccine with 50% efficacy against disease could still be worthwhile to use, even at short-lived natural and vaccine-induced protections. Smaller outbreaks could continue even with vaccines, but enforced population-wide physical distancing measures might no longer be required. Voluntary physical distancing as a sole strategy risks large initial epidemic peaks, unless accompanied by highly effective immunisation. Compared with no vaccination, introducing vaccination leads to positive net monetary value across physical distancing scenarios in the best-case scenario from the health-care perspective, subject to the long-run vaccine price and cost-effectiveness of other treatments (eg, new drugs). The positive net monetary value in the worst-case scenario depends on the assumed vaccine price, discount rate, the monetary value assigned to quality-adjusted life-years, and continued physical distancing. The net monetary value of immunisation decreases if natural immunity is long or vaccine-induced protection is short. Intermittent periods of increased physical distancing lead to negative net benefits from the perspective of the wider economy if the daily national income losses are persistent and larger than under less strict physical distancing scenarios.

**Implications of all the available evidence**

Our model findings highlight the substantial health and economic value of introducing SARS-CoV-2 vaccination alongside physical distancing measures to control the COVID-19 pandemic in the UK, with vaccines likely to achieve good value for money. Despite the many uncertainties, continued physical distancing might be needed to reduce community transmission until high population-wide coverage is achieved with vaccines that provide long-lasting protection against both disease and infection. Ongoing transmission is also expected in an intermediate scenario with partial efficacy against infection. Our study provides the first broad health-economic insights about possible future scenarios in a post-vaccination era, and the value of vaccines will depend on other socioeconomic and health-related policies and population behaviours.

In terms of minimising health losses and economic harm, apart from continued non-pharmaceutical interventions such as physical distancing and extended testing and tracing for SARS-CoV-2 infection, effective and safe pharmaceutical interventions will be required. Vaccines provide a potential control strategy not requiring recurring lockdowns or extensive testing. As of March 12, 2021, WHO is tracking 182 vaccine candidates in preclinical evaluation and 81 candidate vaccines in clinical trials, of which 21 are in phase-3 trials.^7^ The first COVID-19 vaccine was authorised for temporary supply in the UK on Dec 2, 2020, with vaccination rollout starting on Dec 8, 2020.^8^, ^9^ Further vaccines have since been authorised, and the UK has signed agreements for at least seven vaccine candidates and co-funded clinical trials.^9^ Therefore, we aimed to explore the health and economic value of introducing a SARS-CoV-2 immunisation programme in the UK. Given the unknown characteristics of the longer-term epidemiology of COVID-19, we focused on the wider impact of vaccination alongside physical distancing scenarios, as the value of vaccines will depend on other policies and population behaviours (both economic and health related).

## Methods

### Epidemiological model

We used CovidM, an age-stratified dynamic transmission model that was developed to explore the effect of COVID-19 and non-pharmaceutical interventions in the UK.^10^ In this susceptible-exposed-infectious-recovered epidemic model, individuals are stratified into 5-year age bands and susceptible individuals can become infected and move into an exposed compartment before becoming infectious and symptomatic. A predefined proportion of individuals will be asymptomatically infected only. Individuals recover but natural protection can wane over time without affecting the assumptions of reinfections. Our estimated cases refer to symptomatic (clinical) cases. The model also tracks COVID-19-related hospital admissions and deaths by age; for more details, see the study by Davies and colleagues.^10^ We also explored updating parameters using data from the UK (appendix pp 1–4).

We extended the compartmental model to investigate the health impact of an immunisation programme and the economic value of SARS-CoV-2 vaccination in the UK. Individuals who have been vaccinated move into separate compartments, assuming either total protection or no protection from infection and exponential waning of vaccine-induced protection over time; both of these assumptions are conservative given that vaccine trials have been done in settings with high force of infection^9^ and duration of vaccine protection remains unclear.^11^, ^12^ The model structure was further adapted to also allow for protection against disease but not infection. We added demography in terms of births and (disease-unrelated) deaths, which allows the exploration of outcomes over 10 years (2020–29) to sufficiently capture the potential effects of vaccination.

In late 2020, a new SARS-CoV-2 variant was detected in the UK and was estimated to have increased transmissibility by 43–90% (range of 95% credible intervals [CrIs] 38–130).^13^ Our model starts with a basic reproduction number, *R*_0_, of 2·7 (95% CrI 1·6–3·9), which was based on a previously derived consensus distribution of 11 published estimates.^10^ Hence, the uncertainty interval (UI) of the variation in *R*_0_ in our analysis overlaps with the estimated range of basic reproduction numbers of the new variant.^13^ At the start of the model and for the first 6 months afterwards, five new infections are introduced into the population each day for 7 days in the age groups 20–49 years to represent a low but continuous (undetected) importation of infections initially.^14^ In a sensitivity analysis, we limited the importation rate of new infections to the first 6 months, which would in theory allow elimination.

### Immunisation programme intervention and comparator scenarios

The model compared three main scenarios: a no-vaccination baseline scenario versus two vaccination programmes resembling the worst-case and best-case extremes of a reasonable spectrum of assumptions about vaccine characteristics that were partly informed by phase 3 trial data.^9^, ^15^, ^16^ The worst-case vaccination scenario assumed 50% vaccine efficacy^15^, ^17^ and vaccine-induced protection against disease (ie, symptomatic infection is replaced by asymptomatic infection with 50% decreased transmissibility) of 45-week duration (similar to the assumed duration of natural immunity),^18^ whereas the best-case vaccination scenario assumed 95% vaccine efficacy^8^, ^9^ and vaccine-induced protection against both symptomatic and asymptomatic infection of 3-year duration (assumed similar to the reported duration of immunity for SARS-CoV and MERS).^11^ In both vaccination scenarios, we assumed an uptake of 75% in individuals aged 15 years or older and a targeted vaccination rollout according to the current UK prioritisation advice,^19^ and annual revaccination of 50% in individuals aged 15–64 years and of 75% in individuals aged 65 years or older (similarly to seasonal influenza vaccination). Natural protection from infection was assumed to wane within a mean of 45 weeks in the base case.^18^

In sensitivity analyses, we varied the duration of natural and vaccine-induced protection from 12 weeks to 5 years,^11^ the age groups targeted with vaccination (including vaccinating uniformly across age groups), the initial vaccination rate before revaccination, the revaccination coverage, the timing of the vaccination introduction if delayed until the start of 2022, the vaccination uptake rate after the initial phase and before starting annual re-vaccinations, the impact of the imported cases each month, and the vaccine efficacy against disease or infection according to published clinical trial data.^9^, ^15^, ^16^

We assumed vaccination introduction starting from Dec 8, 2020, with protection assumed to start after 28 days.^8^, ^9^

### Physical distancing scenarios

The model considered the vaccination scenarios with and without periods of increased physical distancing. Physical distancing was implemented by reducing the number of close contacts of individuals at home, work, school, and other locations (such as transport and leisure; appendix p 1) that could lead to transmission events. These effective reductions in transmission can be the result of personal non-pharmaceutical interventions such as keeping a safe physical space from individuals from other households, wearing face coverings (masks), and increased hand hygiene. However, these are less susceptible to government interventions and also have much lower societal costs, so we do not explicitly model their effects. We consistently assumed voluntary physical distancing of the public after the initial outbreak in all scenarios and for the remaining duration of the 10 years of the model, with close contacts being reduced by a third at work, school, and other locations. All physical distancing scenarios also considered summer and winter holidays, during which school contacts were reduced to 0 (appendix p 1). The model made no explicit assumptions about increased mixing between children from different schools and locations during holidays (eg, during play dates).

We explored three groups of physical distancing scenarios: no historical lockdown (a counterfactual scenario), an initial lockdown as observed historically (ie, up to mid-July), and an initial lockdown followed by intermittently occurring increased physical distancing where contact rates are reduced once incidence goes over a certain level (varied in sensitivity analysis to be triggered at daily incidence levels of ten to 100 cases per 100 000 population). The initial lockdown and increased physical distancing result in a similar scaling of the contact matrices (appendix p 1). Contacts are increased again once the daily incidence reaches fewer than 500 cases (about one case per 100 000). All simulations started on Jan 1, 2020, for 10 years to allow exploration of counterfactual scenarios.

### Comparison of estimated with observed hospital admissions

For scenarios with an initial lockdown, the incidence threshold for the initial lockdown and start of voluntary physical distancing was based on aligning the estimated cumulative number of hospital admissions to the observed numbers in the UK as of July 15, 2020 (appendix p 1), with an estimated 8·54% of the population having been infected by mid-July, 2020. Rather than reproducing the first few months of the pandemic precisely, however, we did not fit the model to the full range of data available given the many uncertainties and unknown characteristics of the vaccines and aspects of the COVID-19 epidemiology.

### Health and economic impact

The economic analysis was fully integrated with the epidemiological model. Our main analysis adopted the reference case used to evaluate vaccines in the UK,^20^, ^21^ considering benefits in terms of disease prevented using quality-adjusted life-years (QALYs), and the costs from the perspective of the National Health Service (NHS) over a 10-year timeframe. The main outcome was the net monetary value of each of the 27 combined vaccination and physical distancing scenarios, calculated by expressing the combined health (QALYs) and economic benefits of the interventions in monetary terms: QALYs × (monetary value per QALY) – costs.^22^ Net monetary values can be used to show which intervention minimises losses, since all scenarios have negative net monetary values (ie, they destroy economic value) compared with no pandemic. We assumed a monetary value per QALY of £20 000,^20^, ^21^ which we varied between £0 and £60 000 in a sensitivity analysis.^23^ Positive incremental net monetary values of vaccination against no vaccination thus indicate results within the chosen cost-effectiveness threshold (eg, £20 000 per QALY), suggesting that the vaccination or physical distancing scenarios considered are cost-effective. Negative incremental net monetary values indicate results above the cost-effectiveness threshold, suggesting that the scenarios considered are not cost-effective and are dominated by the comparator because of either minimal QALY gains at excessive additional costs or excessive QALY losses being insufficiently compensated for by minimal cost savings.^22^ Future costs and QALYs were discounted at 3·5% annually,^20^, ^21^ and we explored varying the discount rate between 0% and 10% in a sensitivity analysis.^20^, ^23^

To assess the health impact of the interventions, we considered the QALYs lost by symptomatic cases, non-fatal hospital admissions, intensive care unit (ICU) survivors, post-acute (long) COVID, adverse events following immunisation, and premature fatalities due to COVID-19 (appendix pp 8–9). For the costs from the NHS perspective, we considered the expenditures on hospital admissions (ICU and non-ICU), enhanced personal protective equipment, visits to general practitioners, remote helpline calls, adverse events following immunisation, vaccine administrations, and vaccine costs (appendix pp 9–11). We assumed a conservative long-run cost per vaccine dose of £15 (based on the first authorised vaccine in the UK; appendix p 10), which we varied in sensitivity analyses from £0 (ie, without assigning financial cost to the vaccine doses) to £50. Additional costs of setting up a delivery programme have not been included other than a public tender for ultra-low temperature freezers required by the first authorised vaccine (appendix pp 10–11). In addition, the vaccination scenarios included the public expenditures on subsidising the development of SARS-CoV-2 vaccines with £250 million by the UK Government,^24^ which could be regarded as an extraordinary lump-sum ex-ante premium.

We did an exploratory, secondary analysis that considered the negative impact of physical distancing on the wider national economy, and the incremental benefit of introducing vaccination on that impact. This analysis can be used as a framework until more is known about the vaccines. We approximated the costs of physical distancing in terms of losses to the UK's GDP, which is a monetary output measure of all goods and services produced in a country during a specific period. We assumed that daily GDP was £5757 million, based on the seasonally adjusted GDP in the fourth quarter of 2019 of £523 917 million,^25^ and we assumed national income losses from physical distancing and potentially other measures aiming to reduce transmission. GDP losses were conservatively assumed at the equivalent of 2% of daily GDP (ie, around £115·1 million) for each day COVID-19 incidence exceeded 1000 new reported cases while only voluntary physical distancing was in place (similar to the largest pre-COVID-19 reduction since 1997; the 1000 new reported cases were approximated by the numbers in mid-March, 2020).^5^ In addition, losses on days of the scenarios with an initial lockdown and subsequent increased physical distancing were evaluated against the same threshold. However, as the daily GDP loss might differ according to the physical distancing scenario (eg, losses could be higher when increased physical distancing is triggered by a lower incidence threshold), for scenarios with increased physical distancing, we additionally explored higher losses between the equivalent of 2% and 15% of daily GDP whenever increased physical distancing was imposed (including the fall in GDP of 5·8% over March, 2020, and the forecasted 11% decrease in 2020–21).^5^, ^6^ We did not add health sector costs estimated under the NHS perspective or productivity losses in COVID-19 cases to these wider economic costs, to avoid double counting. However, we considered QALY losses for this wider perspective, too, and presented results in terms of the net monetary value.

To account for parameter uncertainty, we ran the epidemiological model deterministically with *R*_0_ values of 2·7 (the base case) as well as 1·6 and 3·9.^10^ The economic model used a probabilistic sensitivity analysis with 1000 iterations using Monte Carlo sampling (appendix p 8).

### Role of the funding source

The funders of the study had no role in study design; data collection, analysis, and interpretation; preparation of the manuscript; or the decision to publish.

## Results

**Figure 1:**
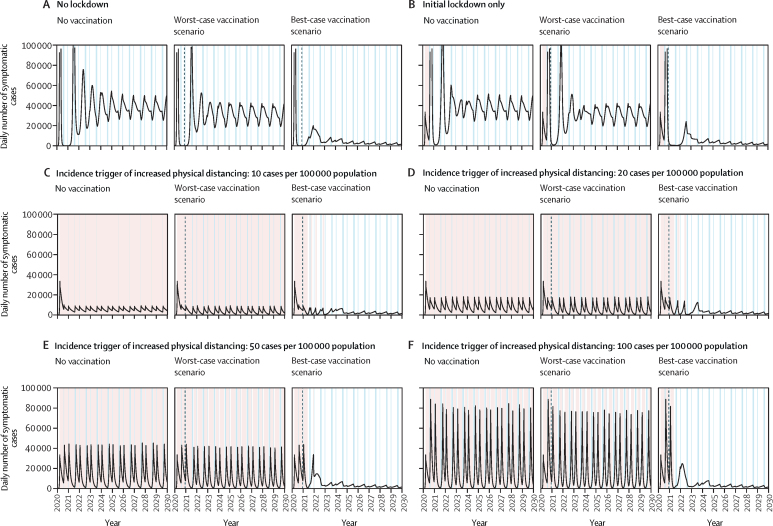
Epidemiological impact of SARS-CoV-2 vaccination in the UK over 10 years Time-series of the daily number of symptomatic cases of COVID-19 in the UK for selected physical distancing scenarios and the three targeted vaccination scenarios (ages 15 years and older). Summer and winter holidays are shown in light blue and periods of increased physical distancing and initial lockdowns in light red. In vaccination scenarios, the dashed vertical line indicates the beginning of the vaccination programme. The *x*-axis starts on Jan 1, 2020. The *y*-axis is truncated at 100 000 cases daily to allow meaningful visual comparisons across panels. Not all physical distancing scenarios are shown for ease of presentation.

**Figure 2:**
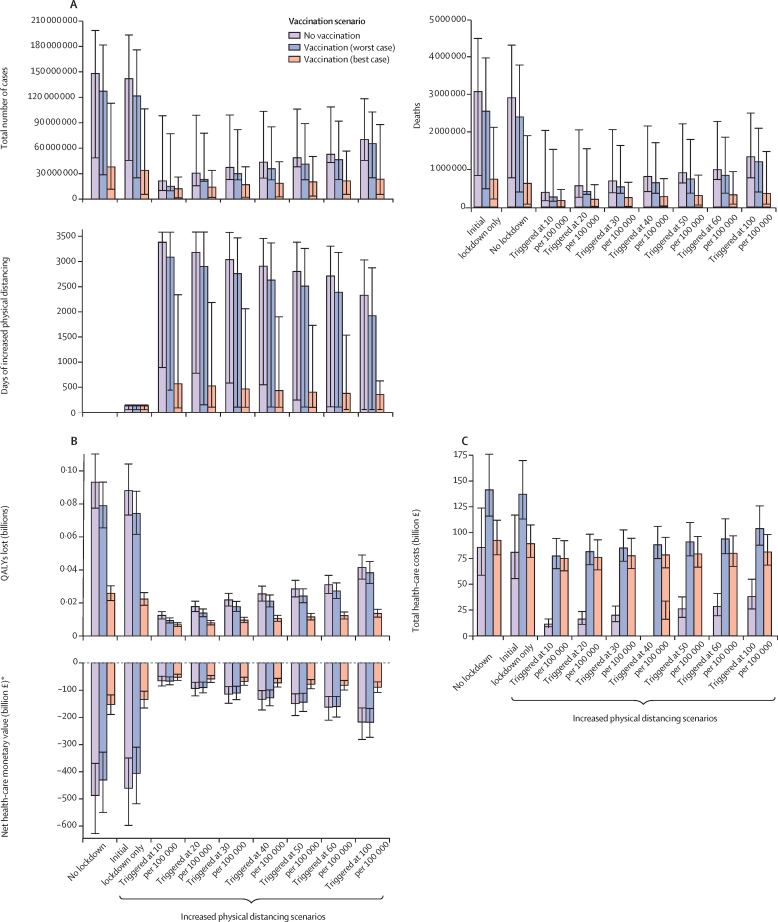
Health and economic impact of SARS-CoV-2 vaccination in the UK Results show impacts over 10 years for different physical distancing scenarios and the three vaccination scenarios. Panel A shows the estimated numbers of cases, deaths, and days of increased physical distancing; panel B shows the total costs and QALYs lost; and panel C shows the net monetary value as costs due to COVID-19 from a health-care perspective. The uncertainty in panel A reflects the values of the 95% credible interval of *R*_0_ values used in the epidemiological model, whereas the uncertainty in panel B reflects the 95% credible interval of the Monte Carlo sampling. QALYs=quality-adjusted life-years. *Net monetary values are negative due to health losses and costs from COVID-19.

An unmitigated epidemic without an initial lockdown is expected to have led to high incidence of infection in the initial outbreak (figure 1A), with smaller but recurring annual outbreaks due to varying contact rates and transmission during holidays, and loss of natural immunity and births replenishing the susceptible population. By contrast, implementing an initial lockdown as observed historically moves the high burden into the second peak (figure 1B), with little change in the overall burden as compared with no lockdown over a 10-year period (figure 2A). Intermittent periods of increased physical distancing without vaccination (baseline scenario) could change the height of future peaks and split up the epidemic into two smaller outbreaks each year (figure 1C–F), with a consequent reduction in burden (figure 2A).

In the worst-case vaccination scenarios, introducing vaccination could lower and delay the peaks of future outbreaks, which is most apparent in the no lockdown and initial lockdown only scenarios (figure 1). Vaccines that are highly effective against disease and infection could reduce transmission to its lowest levels in the model within 3–4 years without further periods of increased physical distancing, while SARS-CoV-2 is likely to become endemic (figure 1). Ongoing transmission is also expected in intermediate scenarios with vaccine efficacy against disease and infection similar to the published clinical trial data (appendix pp 16–17). A targeted vaccination strategy is more effective at reducing case numbers than vaccinating uniformly, even though the disease dynamics are similar (appendix p 18).

The 10-year burden of an unmitigated epidemic in the UK (the counterfactual scenario) was estimated as 148·0 million (95% UI 48·5–198·8) cases, 3·1 million (0·84–4·5) deaths, and 93·1 million (77·5–110·2 million) QALYs lost (figure 2; appendix pp 11–13). Combined with periods of increased physical distancing, these numbers can be reduced over the same time period to 21·4–70·2 million cases, 0·39–1·3 million deaths, and 12·4–41·5 million QALYs lost. Introducing vaccination is estimated to reduce these numbers further to 12·1–65·4 million cases, 0·17–1·21 million deaths, and 6·8–38·3 million QALYs lost. The estimated number of days in lockdown or with increased physical distancing is substantial, unless vaccines with high efficacy and long-lasting protection against infection become available (figure 2; appendix pp 11–12).

From the health-care perspective, the total costs of the COVID-19 pandemic in the UK over 10 years in the unmitigated scenario without vaccination or increased physical distancing amount to £85·6 billion (95% UI 58·7 to 123·8) compared with a situation where the COVID-19 pandemic had never happened (figure 2; appendix pp 12–14). Without introducing vaccination, implementation of increased physical distancing triggered at different incidence levels decreases the costs by £4·7 billion up to £74·3 billion. Depending on the physical distancing scenario, introducing vaccination increases total costs by £6·8–66·0 billion (figure 2B; appendix pp 12–14).

**Figure 3:**
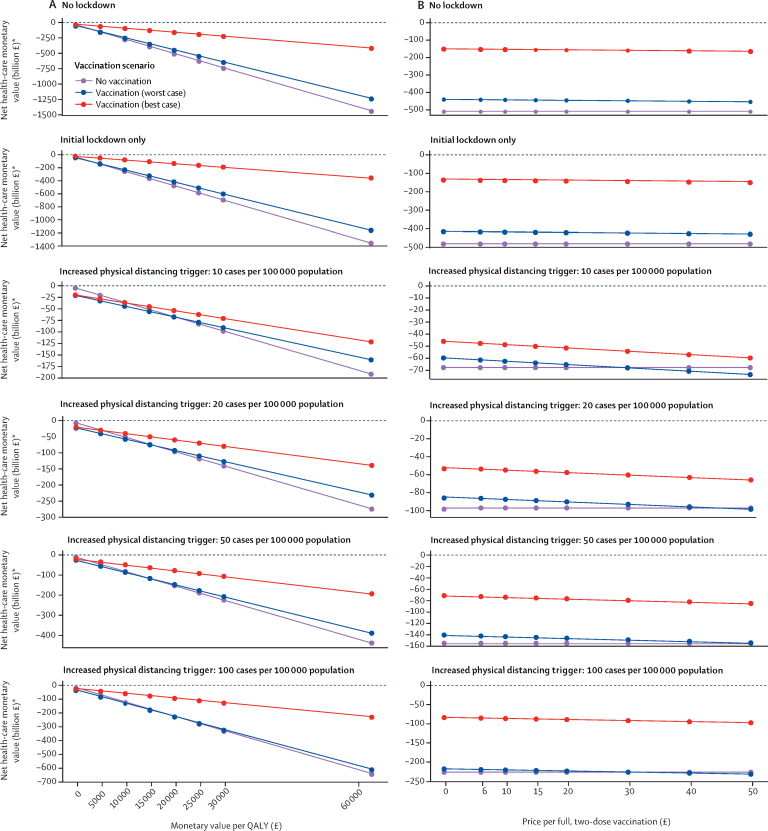
Value of QALYs and COVID-19 vaccine prices Sensitivity analysis on the monetary value per QALY (A) and the price per vaccinated individual, assuming two doses (B), for selected physical distancing scenarios and the three vaccination scenarios. Losses are considered from a health-care perspective. Not all physical distancing scenarios are shown for ease of presentation. QALY=quality-adjusted life-year. *Net monetary values are negative due to health losses and costs from COVID-19.

When considering health outcomes and costs through the net monetary value, losses from COVID-19 were highest for the unmitigated scenario, at £487 billion (95% UI 369–632; figure 2C). Compared with no vaccination, introducing vaccination leads to positive incremental net monetary values in the best-case scenario, ranging from £12·0 billion to £334·7 billion across physical distancing scenarios; however, in the worst-case scenario, incremental net monetary values ranged between –£1·1 billion and £56·9 billion (figure 2C; appendix pp 12–13). Incremental net monetary values were positive in all scenarios at a higher monetary value of a QALY (figure 3A), lower vaccine prices (figure 3B), or lower discount rates (appendix p 19).

**Figure 4:**
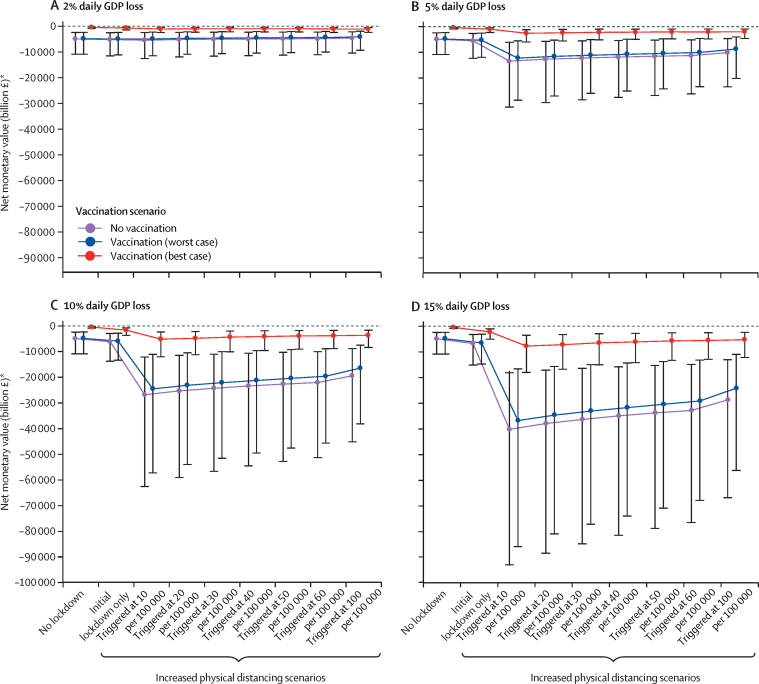
Wider economic impact of SARS-CoV-2 vaccination The potential wider economic impact of vaccination in terms of net monetary values for different (proportions of) daily GDP loss during different physical distancing scenarios. Uncertainty reflects the 95% credible interval of the Monte Carlo sampling. GDP=gross domestic product. *Net monetary values are negative due to health losses and costs from COVID-19.

The incremental net monetary value of introducing vaccination versus no vaccination is always positive across physical distancing scenarios from a wider economic perspective (figure 4). Greater losses of national income are expected with a lower (ie, stricter) incidence-rate threshold for physical distancing, leading to greater absolute net monetary losses from COVID-19 (figure 4).

**Figure 5:**
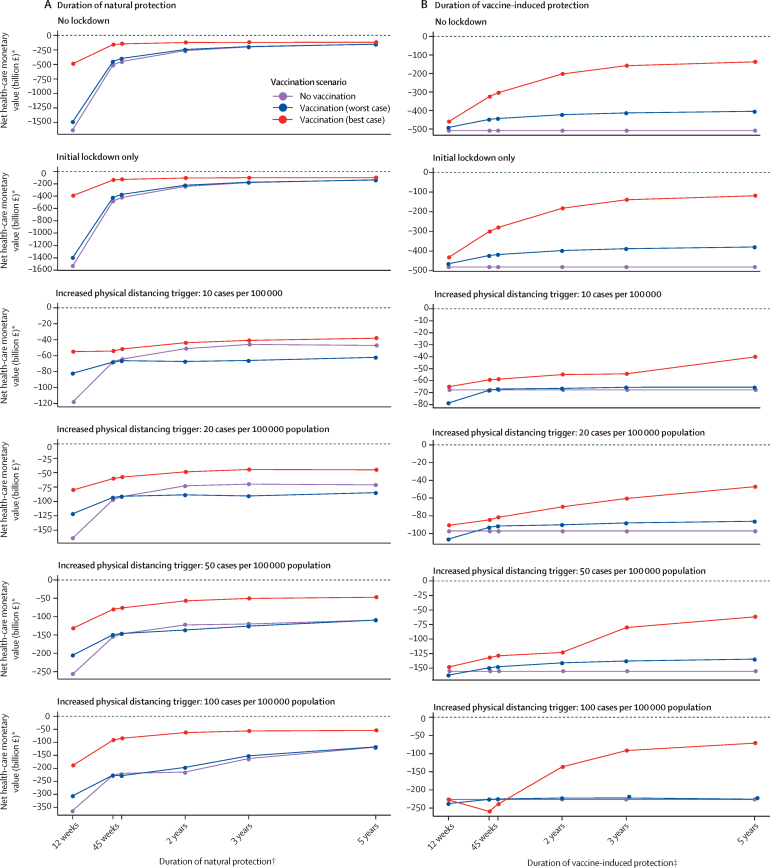
Economic impact of natural and vaccine-induced protection Sensitivity analysis on the economic impact of duration of natural protection (A) and vaccine-induced protection (B) against SARS-CoV-2 infection for selected physical distancing scenarios and the three vaccination scenarios. Losses are considered from a health-care perspective. *Net monetary values are negative due to health losses and costs from COVID-19. †Vaccine-induced protection kept at 45 weeks (worst-case vaccination scenario) and 3 years (best-case vaccination scenario). ‡Natural protection kept at 45 weeks

In sensitivity analyses, we found that with a longer duration of natural immunity, the incremental economic value of immunisation decreases, potentially becoming lower than no vaccination for long durations of natural immunity and 50% vaccine efficacy against disease (worse-case scenario; figure 5A). Similarly, with a longer duration of vaccine-induced protection against disease or infection, the economic value of vaccination compared with no vaccination increases (figure 5B). In the absence of reliable data, however, these findings merely illustrate the sensitivity of results to immunity assumptions, and the level of cross-protection to novel variants remains unclear (and thus supports assuming a shorter duration of immunity until more data become available). If the introduction of safe and effective vaccination had been delayed beyond December, 2020, the economic value was estimated to decrease in most scenarios (with a few exceptions at very strict physical distancing levels and depending on the size of the epidemic peak at the time of vaccine introduction; appendix p 20). The efficiency in terms of incremental net monetary value gained per vaccinated individual increased when targeting vaccines according to prioritisation groups and when vaccines are safe and effective to use in ages younger than 15 years; vaccinating uniformly across age groups was most efficient in terms of incremental net monetary value gained in individuals aged 65 years or older (appendix p 21). Increasing revaccination coverage increased the net monetary value in the best-case scenario only (appendix p 22). Findings were robust against the assumed initial daily vaccination rate before annual revaccination (appendix p 23) and the monthly infection importation rate (appendix p 24).

## Discussion

This study explored the health and economic impact of introducing a SARS-CoV-2 vaccination programme in the UK. Our model findings show that highly effective vaccines with long-lasting protection against infection can reduce transmission over the next 10 years without recurring periods of increased physical distancing, while SARS-CoV-2 is likely to become endemic. Compared with no vaccination, introducing vaccination leads to positive net monetary value across physical distancing scenarios in the best-case scenario. Even in a worst-case scenario in which SARS-CoV-2 vaccines have 50% efficacy and provide only short-lived protection against disease, their use would still offer overall benefit at lower prices than £15 per dose, given the large health and economic costs of COVID-19. The value of the vaccine to the UK alone could be in the billions and possibly trillions of pounds. This indicates that investments in SARS-CoV-2 vaccination have been worthwhile to accelerate the development of vaccine candidates at an unprecedented speed,^1^, ^26^ and to make the vaccines available at accessible prices for both the UK and other countries, instead of competing at premium monopoly prices.

Within the framework of the analysis, our model results indicate the continued importance of physical distancing to reduce community transmission in the future, until population-wide vaccine coverage is sufficiently high. The analysis assumed a reduction of a third in non-home contacts in all scenarios that could lead to transmission events after the initial outbreak, due to people being more likely to continue working flexibly, businesses continuing to impose lower capacity limits on building occupancy, and continued precautionary behaviour by the public after more than a year of the pandemic, among other reasons. The value of periods of increased physical distancing in the worst-case scenario is even greater than reflected in the total QALYs and costs averted, since it splits annual epidemics into two separate peaks, which reduces the overall size of the peaks and hence the risk of exceeding hospital capacity. However, the intensity and economic cost of these subsequent periods of increased physical distancing might be less than those of the initial lockdowns, if they can be combined with other measures such as effective testing and contact tracing, improvements of treatment, better availability of personal protective equipment, and precautionary behaviour such as wearing face coverings and handwashing by the general population.

To our knowledge, this study is one of the first economic evaluations focusing on SARS-CoV-2 vaccination,^27^, ^28^ and using established reference case methods.^20^, ^21^ A few studies model the dynamics of SARS-CoV-2 vaccination (see, for example, Saad-Roy and colleagues^29^). Arguably, a conventional cost-effectiveness framework has its limitations during a pandemic: the monetary value per QALY could be different to the normal decision rules used in the UK given that (1) the value of alternative hospital admissions forgone when admitting COVID-19 cases (ie, the opportunity costs of COVID-19 hospitalisations) is unclear, because alternative admissions being displaced are less well understood during a pandemic than, for example, during times of usual winter bed pressures;^30^ (2) we quantified the reduction in health loss and people generally prefer to avoid losses (loss aversion);^31^ and (3) the health opportunity costs will change with COVID-19 given that the overall health sector budget might shift and COVID-19 will have an impact on the efficiency of other health-care interventions. Some argue that a higher discount rate could be justified given that policy decisions before the emergence of SARS-CoV-2 imply a relatively low value was placed on future non-influenza pandemics.^32^ We also highlight the potential magnitude of broader societal benefit, but given that our methods are simple, and the need for further macroeconomic modelling, we did not explore other future research avenues such as which vaccine product provides best value for money using threshold pricing against a cost-effectiveness threshold or net societal benefit. Likewise, our modelling framework enables future research on the relative value of different observed combinations of vaccine characteristics, physical distancing scenarios, and vaccination strategies potentially including paediatric vaccination and revaccination, once more long-term real-world data have become available.

Our study has various strengths and limitations. We extended an age-structured dynamic-transmission model that was previously used to inform UK policies on introducing non-pharmaceutical interventions to provide early insights into the health and economic value of SARS-CoV-2 vaccination in the UK. Given the many uncertainties of the vaccine candidates and the longer-term COVID-19 epidemiology, however, rather than reproduce the first few months of the pandemic precisely, we chose not to fit the model to the full range of data available. Modelling the counterfactual no-lockdown scenarios provide some useful context for policy makers on what has been observed in other jurisdictions outside the UK. Furthermore, the primary endpoint of many clinical trials for vaccines so far has focused on efficacy against symptomatic disease instead of infection, which can reduce the disease burden but might not prevent infection. When using a high *R*_0_ of 3·9, possibly corresponding to a scenario in which high-transmission variants have replaced original variants, the impact of vaccination is similar to the base-case analysis and does not change conclusions qualitatively. We also did not account for different risks of infection or infectivity by occupation or risk-group other than by age, and we did not include therapeutic pharmaceutical interventions that might not prevent transmission given our focus on vaccines (and which are unlikely to alter broad conclusions). Thus, the main aim of our analysis was to generate qualitative insights, not precise numbers.^33^

In an exploratory secondary analysis, our study also looked at the population-level trade-off in terms of health gains from disease averted versus costs to the economy. Although the modelled scenarios of physical distancing might not predict future disease dynamics for the next decade, physical distancing itself was not the focus of this study. However, ignoring the wider economic impact of physical distancing risks distorting conclusions as a lockdown might indeed help to reduce the health burden and health-care costs and thus look highly cost-effective from a health-care perspective, but it will harm the wider economy and society from a broader perspective. We also did not account for the economic impact of physical distancing causing long-term harm (such as reducing productivity) or conversely being mitigated by longer-term structural change (such as shifts in employment towards economic sectors more resilient to physical distancing measures), that the actual GDP impact during the initial lockdown was mitigated in the UK by government actions that bear future cost implications, and that governments might become more efficient at minimising health loss versus economic trade-offs over time. Similarly, we did not account for future economic shocks. Although the analysis considered the impact for the UK population with 66·4 million individuals in England, Wales, Scotland, and Northern Ireland, the policy measures of physical distancing differ slightly between the four. Also, the study focused on the direct impact of COVID-19 and did not address growing concerns about inequality.

Additional factors that were not considered in this analysis might become important in the future, including enhanced testing programmes. Some of these effects might have been included implicitly in our study by assuming long-term lower contact patterns of individuals following the first epidemic peak. Our economic analysis is also conservative in its input parameters, informing costs and quality-of-life values largely from other respiratory infections, but attempting to account for the impact of post-acute (long) COVID on quality of life.^34^ Moreover, our study ignored indirect effects such as the health opportunity costs for displaced patients without COVID-19, potentially increased anxiety induced by COVID-19 in otherwise healthy individuals, excess mortality, the longer-term impact of both COVID-19 care and COVID-19-related physical distancing on other treatments (eg, delays in cancer care^35^), and other changes in demography (eg, on birth rates and perinatal outcomes). Similarly, the analysis assumes the same level of indirect herd effects from vaccines over 10 years, and might underestimate the impact of vaccination beyond 10 years. Both the COVID-19 pandemic and the SARS-CoV-2 vaccination programme might also disrupt health-care service delivery of other vaccination programmes,^36^, ^37^, ^38^ and possibly the disease dynamics of other close-contact infections.^39^ We were also unable to provide accurate estimates on the wider social opportunity costs arising from the longer-term impact on mental health, household finances, and education both at an individual and a societal level.^32^ Some of these difficulties stem from estimating how COVID-19 interacts with feedback loops in the health system as an external shock and will need to be quantified later. Including these effects, however, is likely to increase the value of vaccination.

In conclusion, our findings highlight the health and economic value of introducing SARS-CoV-2 vaccination to control the COVID-19 pandemic. Continued physical distancing might be needed to reduce community transmission until high population-wide coverage is achieved with vaccines that provide long-lasting protection against both disease and infection. Our study provides broad insights rather than precise quantitative projections given the many uncertainties and unknown characteristics of the vaccine candidates and aspects of the long-term COVID-19 epidemiology, and the value of vaccines will ultimately depend on other policies and population behaviours (both economic and health related).

## Data sharing

We have used publicly available data to inform this study, cited in the reference list or in the appendix (pp 28–31). The CovidM modelling framework used has been published previously and is available on the CMMID COVID-19 GitHub page.
