# Correction to *Lancet Infect Dis* 2021; 21: 962–74

**DOI:** 10.1016/S1473-3099(21)00478-3

**Published:** 2021-10

*Sandmann FG, Davies NG, Vassall A, et al. The potential health and economic value of SARS-CoV-2 vaccination alongside physical distancing in the UK: a transmission model-based future scenario analysis and economic evaluation.* Lancet Infect Dis *2021; **21:** 962–74*—In this Article, the age group included in the vaccination programme should have been described as individuals aged 20 years or older. As a result, the text has been corrected in the Summary and Methods sections, the figure 1 legend, and the appendix. Furthermore, the y-axis scale of figure 4 has been corrected. These corrections have been made to the online version as of Aug 9, 2021.

