# Supplementary material for: The potential health and economic value of SARS-CoV-2 vaccination alongside physical distancing in the UK: a transmission model-based future scenario analysis and economic evaluation
Source: Lancet Infect Dis. 2021 Jul;21(7):962–74. doi: 10.1016/S1473-3099(21)00079-7 (PMC7972313; doi:10.1016/S1473-3099(21)00079-7)
Supplement: Supplementary appendix [file mmc1.pdf]

THE LANCET
Infectious Diseases

Supplementary appendix

This appendix formed part of the original submission and has been peer reviewed. We post it as supplied by the authors.

Supplement to: Sandmann FG, Davies NG, Vassall A, et al. The potential health and economic value of SARS-CoV-2 vaccination alongside physical distancing in the UK: a transmission model-based future scenario analysis and economic evaluation. *Lancet Infect Dis* 2021; published online March 18. https://doi.org/10.1016/S1473-3099(21)00079-7.

Supplementary material

Physical distancing scenarios 1
Supplementary Table 1: Reduced contacts between individuals who are physically distancing 1
Burden estimation 2
Supplementary Table 2: Rationale for assumptions on vaccine effectiveness in the range of vaccination scenarios 2
Supplementary Table 3: Input parameters for the epidemiological model 6
Supplementary Table 4. Input data of infection-fatality-rate 7
QALY loss input data 8
Supplementary Table 5: Input parameters in terms of QALYs 8
Healthcare cost input data 9
Supplementary Table 6: Input parameters in terms of costs 10
Additional results 11
Supplementary Table 7: Results in terms of cases, deaths, and days spent in physical distancing per physical distancing scenario and vaccination scenario 11
Supplementary Table 8: Results in terms of QALYs, costs, and net monetary value from the healthcare perspective per physical distancing and vaccination scenario 12
Supplementary Table 9: Results in terms of total and incremental QALYs and costs, and the incremental cost-effectiveness ratio, from the healthcare perspective per physical distancing and vaccination scenario 14
Sensitivity analyses 15
Supplementary Figure 1: Epidemiological impact of vaccination effectiveness (AstraZeneca-Oxford University vaccine) 16
Supplementary Figure 2: Epidemiological impact of vaccination effectiveness (Pfizer-BioNTech vaccine) 17
Supplementary Figure 3: Vaccinating uniformly 18
Supplementary Figure 4: Sensitivity analysis on the discount rate 19
Supplementary Figure 5: Timing of vaccination 20
Supplementary Figure 6: Age group of vaccination and target groups 21
Supplementary Figure 7: Re-vaccination coverage level 22
Supplementary Figure 8: Vaccination uptake rate 23
Supplementary Figure 9: Epidemiological impact of imported cases 24
CHEERS checklist 25
References 28
Centre for Mathematical Modelling of Infectious Disease COVID-19 working group 32

Supplementary Material

Physical distancing scenarios

For scenarios with an initial lockdown, the incidence threshold for the initial lockdown and physical distancing was based on aligning the results for the estimated numbers of COVID-19 related hospitalisations and deaths to the observed numbers in the UK. As of 15 July 2020, there were 130,472 recorded admissions and 41,035 deaths due to COVID-19 in the UK.[1] The model most closely resembled these values when going into lockdown once the incidence reached 30 cases per 100,000 population and using the values shown in Supplementary Table 1, with an estimated 136,500 admissions and 37,128 deaths by 15 July 2020.

There are many unknowns surrounding the characteristics of the vaccine candidates, uncertain aspects of the longer-term COVID-19 epidemiology, what measures will be put in place in future, and the COVID-19-specific impact on costs and QALYs. However, in this economic evaluation a higher value was placed on approaching the absolute numbers as observed historically rather than closely resembling the observed disease dynamics, which are difficult to predict in future, too. Although the modelled scenarios of physical distancing may not perfectly predict future disease dynamics for the next decade, physical distancing itself was not the focus of this study evaluating the impact of SARS-CoV-2 vaccination. However, ignoring the wider economic impact of physical distancing risks distorting conclusions as an indefinite lockdown may indeed help reduce the health burden to a minimum and at minimal healthcare costs, but at substantial harm to the wider economy and society.

Supplementary Table 1: Reduced contacts between individuals who are physically distancing; these values were used to scale the underlying contact matrices obtained from POLYMOD.[2]

	home	work	school	other
initially	1.0	1.0	1.0	1.0
voluntary physical distancing after the first (and subsequent) outbreak(s) (once daily incidence <500 cases, about <1/100,000 cases)	1.0	0.67	0.67	0.67
summer/winter holidays	1.0	0.67	0.0	0.67
initial lockdown (all scenarios except the "no lockdown" one)	0.9	0.1	0.1	0.1
subsequent physical distancing (triggered at incidence rates of 10-60/100,000 cases; released again once daily incidence <500 cases)	1.0	0.1	0.1	0.1

Note: The relative scaling of contacts during the initial lockdown was chosen to achieve a 75% reduction of Rt as observed historically in the UK in 2020,[3] while closely matching observed admissions in the UK in mid-July 2020. Future summer and winter holidays were chosen in line with the dates of school holidays in England in 2020/2021.

Burden estimation

The first two COVID-19 vaccines to have dossiers submitted to authorise supply in the UK market use an mRNA platform (Pfizer, BioNTech) and an adenovirus vector (AstraZeneca, Oxford University), respectively.[4,5] The trial data for the Pfizer-BioNTech vaccine demonstrated vaccine efficacy in adults aged 16+ years of 95.0% (95% credible interval of 90.3% to 97.6%), and in individuals aged 65+ years the vaccine efficacy was 94.7% (two-sided 95% confidence interval of 66.7% to 99.9%).[6-8] Of note, the dossier submitted to the FDA also reported a vaccine efficacy of 52.4% (29.5% to 68.4%) after the first dose and before the second dose, indicating some protection against disease after one dose.[7,8] High vaccine efficacy has also been reported for other vaccines,[5,9] with the AstraZeneza-Oxford University vaccine showing a vaccine efficacy in adults aged 18+ years of up to 90.0% (67.4% to 97.0%) in participants who received a low dose followed by a standard dose of the vaccine.[10] Preliminary results of the trial also reported protection against asymptomatic infection with a vaccine efficacy of 58.9% (95%-CI 1.0% to 82.9%).[10] A third vaccine using an mRNA platform has been authorised on 08 January 2021 (Moderna), which has lower cold chain requirements than the Pfizer-BioNTech vaccine at an overall vaccine efficacy in adults aged 18+ years of 94.1% (95% credible interval of 89.3% to 96.8%).[11]

Based on these initial trial data we thus evaluated a vaccine that can be given to all individuals aged 15+ years, which is reflective of (1) the current guidance of vaccination prioritisation for the rollout in the UK that targets vaccinating clinically extremely vulnerable individuals from 16+ years;[4] (2) the paediatric vaccine trials that are planned and are likely to get a push given that younger ages are becoming increasingly linked to transmission; and (3) the timeframe of our analysis spanning the next 10 years. Note that the transmission model is stratified into 16 age bands of 5 years each (starting from 0-4 years and ending at 75+ years), and for technical reasons thus the strategies started at 15+ years. However, we have provided additional sensitivity analyses that explored alternative vaccination strategies starting vaccinating from 20+ years or indeed 10+ years (should the future licensure permit it); Supplementary Figure 6. Furthermore, it is important to note that while the AstraZeneca vaccine trials found similar efficacy when participants who were seropositive were included, most of the clinical trials published so far have been conducted in COVID-naïve individuals, and the real-world vaccine effectiveness will need to be established later. However, the true vaccine effectiveness will fall within the extreme range of best-case to worst-case scenarios we have considered in this study.

Of note, while any infection event (i.e., involving either symptomatic or asymptomatic disease progression) can be an important driver of transmission between individuals, the primary endpoint of many clinical trials for vaccines has been "symptomatic infection" instead of "asymptomatic infection". There is no evidence that any COVID-19 vaccine blocks transmission in vaccinees who are infected. However, they may contribute to decreasing transmission by preventing infection in the first place and/or decreasing the severity (and hence transmissibility) of infections. This is an important semantic distinction helping to clarify the working of the COVID-19 vaccines and the focus of the clinical trial data published so far.

Supplementary Table 2: Rationale for assumptions on vaccine effectiveness in the range of vaccination scenarios		
Parameter	Assumption	Rationale
Vaccine effectiveness in base case analysis: best-case scenario	95% against infection<br><br>0% additional protection against disease	Based on phase 3 trial data of multiple COVID-19 vaccines that consistently reported high vaccine efficacy against virologically-confirmed COVID-19 disease of up to 95%.[8]for BNT162b2 and 90% for AZD1222[5,10]<br>In this optimistic scenario, we assume that disease averted also eliminates the possibility of infectiousness. There is some evidence of reduced viral load and protection against infection from animal studies of COVID-19 vaccines.[12,13]<br>Note that we assumed 0% protection against disease for the remaining 5%, but if the vaccines are able to prevent

		disease then the benefits of the best-case scenario are expected to be even slightly higher than we estimated.
Vaccine effectiveness in base case analysis: worst-case scenario	0% against infection<br><br>50% additional protection against disease	Based on (i) the minimum requirements for vaccine licensure as formulated by the FDA;[14], (ii) the reported vaccine efficacy of 52.4% after the first dose and before the second dose for BNT162b2;[8], (iii) and the values usually observed for seasonal influenza vaccines.[15]<br><br>Note that we assumed 0% net protection against infection. This assumes that all disease cases prevented will be converted to asymptomatic infection with half the transmissibility. This is not reflective of pre-clinical trial data or of the AZD1222 trial results that included asymptomatic infection,[10] but in the absence of longer and more robust clinical trials this assumption is more conservative in reflecting a worst-case scenario.
Vaccine effectiveness in sensitivity analysis: AZD1222 (AstraZeneca) vaccine best-case scenario	70% against infection<br><br>70% additional protection against disease (91% overall disease protection)	Based on the upper-bound values of the reported overall vaccine efficacy for AZD1222,[10] and in line with values that may be observed for seasonal influenza vaccines.[15]<br>In the phase 3 trial for AZD1222, 62.1% - 90% efficacy against symptomatic COVID-19 was observed (with the range depending on the dose regimen received), and 3.8% - 58.9% efficacy against asymptomatic infection. Even the lower efficacy against asymptomatic infection (3.8%, and not significant) is likely to be consistent with some protection against infection, since the vaccine will have converted some episodes of symptomatic disease to asymptomatic infection, and hence must additionally prevent asymptomatic infection in order to avoid negative efficacy against asymptomatic infection.<br>Efficacy of 70% against infection and an additional 70% against disease is consistent with reported efficacy of 91% against symptomatic disease, 56% against asymptomatic disease, and asymptomatic infection accounting for 85% of all infections.[a]
Vaccine effectiveness in sensitivity analysis: AZD1222 (AstraZeneca) vaccine worst-case scenario	13% against infection<br><br>56% additional protection against disease (62% overall disease protection)	See above; based on the lower-bound values of the reported overall vaccine efficacy for AZD1222,[10].[15]<br>Efficacy of 13% against infection and an additional 56% against disease is consistent with reported efficacy of 62% against symptomatic disease, 4% against asymptomatic disease, and asymptomatic infection accounting for 85% of all infections.
Vaccine effectiveness in sensitivity analysis: values of BNT162b2 (Pfizer-BioNTech) vaccine	50% against infection<br><br>90% additional protection against disease (95% overall disease protection)	Based on the reported overall vaccine efficacy for BNT162b2 vaccine.[6-8]<br>Efficacy of 50% against infection and an additional 90% against disease is consistent with reported efficacy of 95% against symptomatic disease, 36% against asymptomatic disease, and asymptomatic infection accounting for 85% of all infections.

a: In our model we assume total efficacy against disease; if VE against disease is additive to VE against infection then VE should be lower. To illustrate, suppose asymptomatic infections represent a proportion A of all infections in clinical trials of a vaccine. Further, let V_i and V_d be vaccine efficacy against infection and disease, respectively. If someone who has been successfully immunised receives an infectious dose of SARS-CoV-2 (which would normally cause disease in the person), then there are a number of possibilities:

1. The infection and disease could be both completely prevented: this occurs with probability V_i.
2. The disease could be prevented, but the person still becomes asymptomatically infected: this occurs with probability $(1-V_i) * V_d$.
3. The vaccine could fail to prevent disease: this occurs with probability $(1-V_i) * (1-V_d)$

Suppose a clinical trial reports the following efficacy figures:
- Efficacy against symptomatic COVID-19 = T_s
- Efficacy against asymptomatic SARS-CoV-2 infection = T_a

Then we have $T_s = V_i + (1-V_i) * V_d$ and $T_a = A * V_i - (1-A) * (1-V_i) * V_d$. If A = 85%, V_i = 70% and V_d = 70%, then we get T_s = 91% and T_a = 56%.

Furthermore, we accept that not much is known about the duration of natural immunity to SARS-CoV-2, and there is substantial uncertainty around our base case assumption of 45 weeks. However, a shorter duration of immunity is consistent with current evidence including: (i) short-lived humoral [16] and cell-mediated [17] immunological memory, (ii) studies of other coronaviruses suggesting immunity lasting around 1 year,[18] (iii) evidence of short-lived protection emerging from places like Manaus, Brazil, where ~75% of its population had been estimated to be infected but which continues to see a worsening of the local outbreak (as reported in the media in January 2021), (iv) documented reinfections in cohorts such as healthcare workers,[19] (v) evidence of immune escape from new variants which may continue to emerge in the future. All in all immunity is still poorly understood and requires further research, which is why we have considered a shorter value of 45 weeks in the base case analysis that we contrasted with a wider range of values in a sensitivity analysis.

In line with the provisional vaccination prioritisation strategy in the UK,[4,20] our analysis started vaccinating individuals equivalent to the proportion of care home residents (an assumed 291,000 individuals aged 65+ years),[21] health and social care workers (1.52 million social care workers and 1.31 million NHS workers),[22,23] individuals aged 75+ years, 70+ years and those at high-risk but under 65 years (for which we obtained age-stratified data from Clark et al., 2020),[24] 65+ years, those at moderate risk under 65 years,[24] those aged 60+ years, 55+ years, 50+ years, and then everyone else remaining and aged between 15 and 49 years. In sensitivity analysis we also explored vaccination at different ages, and vaccinating uniformly across all age groups.

We have assumed an initial uptake of 75% in line with recent cross-sectional survey research on the likely uptake of a COVID-19 vaccine in a UK sample,[25] and with re-vaccination coverage of 50% in individuals aged 15-64 years and 75% in adults aged 65+ years (in line with the observed seasonal influenza uptake). In both vaccination scenarios we assumed an initial vaccination rate of 100,000 individuals a day, which was informed by the initially limited supply of 800,000 doses for the vaccination rollout on 08 December of the vaccine authorised first in the UK.[26] After the first prioritisation groups have been vaccinated, we assumed a higher vaccination rate of 200,000 individuals a day based on the total stock of doses procured in contracts of the UK government that suffices to vaccinate each resident of the UK thrice;[5] a total number of three vaccines being authorised so far in the UK in early 2021 with more expected;[5] the total number of 1.18 million GP appointments on a weekday;[27] following the recent amendment of the regulation allowing the administration of vaccines under supervision by non-registered healthcare professionals during a pandemic such as with COVID-19 (regulation 247A);[28] and the observed vaccination rate of 181,300 doses a day based on administering 9,790,576 vaccines over 54 days since start of the rollout on 31 January 2021.[1] Higher and lower vaccination rates were explored in sensitivity analysis. For re-vaccinations, we assumed a lower rate of up to 100,000 individuals daily (which is not reached in the base case with assumed coverage levels of 50% in individuals aged 15-64 years and 75% in older individuals aged 65+ years).

We considered the health burden of COVID-19 and related interventions in terms of symptomatic cases, non-fatal hospitalisations, intensive-care unit (ICU) survivors, adverse-events following immunisation (AEFI), and premature fatalities due to COVID-19. For the healthcare costs, we considered visits to general practitioners, remote helpline calls, hospitalisations (ICU and non-ICU), enhanced personal protective equipment, AEFI, vaccine administrations, and the vaccine costs. We assumed twice the QALY loss and costs for AEFI, and twice the costs of vaccine administration and vaccine dose to reflect a 2-dose vaccine.[5]

Key epidemiological illness parameters were taken from CovidM (Supplementary Table 3),[29] which in turn informed these values by published estimates. For our study, we extended the model to incorporate demography in terms of births and (disease-unrelated) deaths to replenish susceptible individuals, assuming a death rate identical to the birth rate based on 713,000 live births in the UK in 2019,[30] which allows exploring a longer timeframe over ten years (2020-2029). We also updated the proportion of inpatients who were admitted to critical care (0.17) and who died in critical care (0.32) based on a large study from the UK of more than 20,000 inpatients.[31] Similarly, we used the age distribution of hospitalisations to inform the age-dependent proportion of hospital admissions in the UK.[31]

Supplementary Table 3: Input parameters for the epidemiological model			
Parameter	Value	Distribution	Sources
Latent period (E to I_P and E to I_S; days)	4.0	gamma	Davies et al. (2020)[29]
Duration of preclinical infectiousness (I_P to I_C; days)	1.5	gamma	Davies et al. (2020)[29]
Duration of clinical infectiousness (I_C to R; days)	3.5	gamma	Davies et al. (2020)[29]
Duration of subclinical infectiousness (I_S to R; days)	5.0	gamma	Davies et al. (2020)[29]
Incubation period (E to I_C; days)	5.5	derived	dE+dP
Susceptibility to infection on contact	age-dependent	derived	Davies et al. (2020)[29], Davies et al. (2020)[32]
Probability of clinical symptoms on infection for age group i	age-dependent	derived	Davies et al. (2020)[29], Davies et al. (2020)[32]
Relative infectiousness of subclinical cases	50%	fixed	assumed
Delay from onset to hospitalisation (days)	7.0	gamma	Davies et al. (2020)[29]
Duration of hospitalisation in non-ICU bed (days)	8.0	gamma	Davies et al. (2020)[29]
Duration of hospitalisation in ICU bed (days)	10.0	gamma	Davies et al. (2020)[29]
Proportion of hospitalised cases that require critical care	17%	fixed	Docherty et al. (2020)[31]
Proportion of fatal hospitalised cases that require critical care	32%	fixed	Docherty et al. (2020)[31]
Delay from onset to death (days)	22.0	gamma	Davies et al. (2020)[29]
Delay from infection to death (days)	26.0	derived	latent period and delay from onset to death
Birth/deaths annually	713,000	fixed	ONS (2019)[30]
ICU: intensive-care unit, ONS: Office for National Statistics.			

In addition, we informed mortality in individuals aged <75 years per 5-year age-band using ensemble infection-fatality rates based on data on COVID-19 confirmed deaths from 45 countries and 22 seroprevalence studies,[33] while infection-fatality rates in individuals aged ≥75 years were based on the REACT3 study from the UK[34] (Supplementary Table 4). Our results are slightly different from earlier analyses[29] due to using different input parameters for mortality, and the model including waning and demography. Our analysis assumed that input values can be extrapolated for the whole of the UK.

Supplementary Table 4. Input data of infection-fatality-rate.			
Age	Value	95% CrI (lower)	95% CrI (upper)
0-4	0.003%	0.002%	0.003%
5-9	0.001%	0.000%	0.001%
10-14	0.001%	0.001%	0.001%
15-19	0.003%	0.002%	0.003%
20-24	0.006%	0.005%	0.008%
25-29	0.013%	0.011%	0.015%
30-34	0.024%	0.021%	0.028%
35-39	0.040%	0.034%	0.047%
40-44	0.075%	0.064%	0.087%
45-49	0.121%	0.104%	0.140%
50-54	0.207%	0.177%	0.239%
55-59	0.323%	0.277%	0.373%
60-64	0.456%	0.392%	0.527%
65-69	1.075%	0.921%	1.244%
70-74	1.674%	1.435%	1.937%
75+	11.64%	9.22%	14.06%
CrI: Credible interval			

QALY loss input data

Estimates of health-related quality of life associated with having (long-term) COVID-19 that used the preferred instrument in England (the EQ-5D) are still scarce currently. Consequently, the QALY losses were largely informed from previously published values of other respiratory infections (Supplementary Table 5), which may underestimate the health gain from preventing persistent symptoms of COVID-19 lasting several months.[35] Once more information about COVID-19 become available these estimates could be updated later, or indeed further explored more conceptually.

QALYs lost per symptomatic case were based on ILI for 2009 H1N1 pandemic influenza in the UK,[36] while QALY loss per non-fatal hospitalisation were based on participants discharged from a large University hospital in the UK (assuming an impact for the hospital stay and maximum duration post-discharge of 71 days).[37] The resulting QALY loss per hospitalisation due to COVID-19 was estimated to be similar to seasonal influenza (0.0201 vs 0.018, respectively).[38]

QALYs lost per non-fatal ICU stay were based on decrements in quality of life of two studies in ICU survivors from the UK.[39,40] The difference in utility over one year was 0.10 in the study of Griffiths et al. (2013) and ~0.15 in the study by Cuthbertson et al. (2010), but it continued at roughly 0.10-0.15 in year 2.5 and year 5 as reported by Cuthbertson et al. (2010).[40]

QALYs lost from post-acute (long) COVID symptoms were based on the relative ratio of disability weights for post-acute consequences to moderate community cases (0.219/0.051=4.29),[41] which we multiplied with the assumed QALYs lost per symptomatic case (4.29*0.008=0.034 QALYs),[36] and assuming a proportion of 10% of cases experiencing long COVID symptoms.[42] This is a conservative estimate on the impact of the quality-of-life based on the assumed QALYs lost per symptomatic case, and more research is needed on the long-term impact of long COVID. A higher QALY value would result in more favourable results by preventing larger QALY losses.

QALYs lost from adverse events following immunisation (AEFI) were assumed with 1 QALD at a chance of 10%, which is roughly following another study on influenza vaccination.[43] We did not consider longer-term or serious AEFI that may occur but are unknown yet.

QALYs lost per death were based on the most recent life expectancy in the UK as 3-year average over 2017-2019,[44] and adjusted for age- and sex-specific QALY population norms based on the EQ-5D-3L for the UK.[45] We also adjusted for the higher prevalence of comorbidities in individuals most likely to die from COVID-19 if infected,[46] by reducing the QALY norms by an assumed 10% and accounting for an assumed 25% increased risk of non-COVID-19 mortality in these individuals.

To explore parameter uncertainty, we ran the epidemiological model deterministically with R0 values of 2.7 (the base case), 1.6, and 3.9.[29] The economic model obtained 1,000 iterations using Monte Carlo sampling of the input costs and QALYs to obtain a probability distribution of outcomes. We used beta distributions for the utilities and a normal distribution for the estimated QALYs lost due to premature mortality. We explored the uncertainty using values that were ±25% of the mean value (±10% for the QALYs lost due to premature mortality) to make as few assumptions as possible on the data and variance.

Supplementary Table 5: Input parameters in terms of QALYs

Parameter	Value	Distribution	Sources
QALY loss per symptomatic case	0.008	beta	Van Hoek et al. (2011)[36]
QALY loss per non-fatal hospitalisation	0.0201	beta	Halpin et al. (2020)[37]
QALY loss per non-fatal ICU	0.15	beta	Cuthbertson et al. (2010)[40]<br>Griffiths et al. (2013)[39]

QALY loss per post-acute (long) COVID	0.034	beta	Van Hoek et al. (2011)[36]; Salomon et al. (2015); Wyper et al. (2020);[41] Greenhalgh et al. (2020)[42]
QALY loss per AEFI	0.00027	beta	assumed 1 QALD at a chance of 10%[43]
QALY loss per fatality	(differ by age group)	normal	ONS (2020)[44]<br>Ara and Brazier (2010)[45]<br>Briggs (2020)[46,47]
AEFI: adverse events following immunisation, COVID: coronavirus disease, ICU: intensive-care unit, ONS: Office for National Statistics, QALD: quality-adjusted life day, QALY: quality-adjusted life year.			

Healthcare cost input data

For the healthcare perspective, we considered the costs associated with visits to general practitioners (GPs), remote helpline calls, hospitalisations (ICU and non-ICU), enhanced personal protective equipment (PPE), AEFI, vaccine administrations, and the vaccine costs.

Costs per GP visit were based on published unit costs in 2019,[48] while we informed the proportion of 5% physical visits to GP practices based on published data for England from week 9-23.[49] Similarly, remote helpline calls were approximated with calls to NHS111 in England, which were costed using the estimated costs of £12.26 per call in 2011 that we inflated to 2019 (£13.86).[48,50] The proportion of 10% calls was again informed by the published data for England.[49]

Costs per non-ICU hospitalisation and per ICU hospitalisation were based on the NHS reference costs 2018/19.[51] Non-ICU hospitalisations were approximated with ICD-10/HRG codes for other viral pneumonia: J12.8 (Other viral pneumonia), J12.9 (Viral pneumonia, unspecified). The base HRG code per hospitalisation for other viral pneumonia is DZ11 (PD14 for age <=18 years). ICU-hospitalisations were approximated with Adult Critical Care (activity-weighted HRG codes XC01Z- XB07Z) and Paediatric Critical Care (activity-weighted HRG codes XB01Z- XB07Z). The ICU costs are an estimate per bed-day, and we assumed a mean stay of 10 days in ICU.[29] Of note, these hospitalisation costs are slightly lower than those published for the 2009 H1N1 influenza pandemic,[52] which may underestimate the costs per hospitalisation and thus the cost-effectiveness of averting hospitalisations.

Costs per enhanced PPE were based on a previous study on MERS-CoV in 2015,[53] which estimated the additional costs on enhanced PPE equipment (mask, gown, gloves, goggles) at £2.50 per patient visit. Accounting for the additional time of an estimated 15 minutes to put on and take off the PPE as well as disposal plus documentation per patient visit, at 6 visits per patient per day came at additional £29.50 for nurses and £45 for physicians, and the total costs per patient at £119 (uprated to 2019 value at £127.62).[48,53] We conservatively estimated the costs based on the daily estimated number of inpatients, assuming one nurse caring for 8 patients.[54] This may underestimate the true costs of PPE, which would underestimate the cost savings from avoiding hospitalisations.

For the costs per AEFI we followed the assumption made by others to use the costs of 1 GP visit per AE.[48,55] again assuming a chance of 10%.[43] No costs for longer-term/serious AEFI were included.

The costs of vaccine administration were based on the agreed service payment for COVID-19 vaccination of £12.58 per dose (and £25.16 for two doses),[56] which is 25% higher than the current service payment for influenza vaccines of £10.06.[57] Although assumed to be administered via GPs, we did not account for the costs of extraordinary GP visits (i.e., we implicitly assume the vaccine to be administered as part of another visit; this assumption may be challenged given the current advise of physical distancing, and increasing the costs per vaccine was shown to be somewhat sensitive for the worst-case vaccination

scenario depending on the physical distancing scenario in sensitivity analysis). We also assumed administration costs to be the same for all vaccines, which is in line with guidance of NHS England as of mid-January 2021;[58] if one was to assume higher or lower administration costs these could also be added to the price per vaccinated individual (see Figure 3, and Supplementary Table 8).

The cost per vaccine dose were conservatively assumed to be £15 to match reports of the first authorised vaccine in the UK that the government has an agreement with for 40 million doses. We varied the vaccine costs in sensitivity analyses at £0-£50, which reflects the range of prices reported for the vaccines that the UK has signed agreements for (including the reported £6 for 2 doses of the AstraZeneza-Oxford University vaccine that the UK has an agreement with for 100 million doses, and the reported £50 for 2 doses of the Moderna vaccine that the UK has ordered 5 million doses; all of these estimates are only indicative at this time as they are based on wholesale prices negotiated in volume-supply contracts between manufacturers and governments).[5] In addition, the vaccination scenarios included the public expenditures on subsidising the development of SARS-CoV-2 vaccines with £250 million by the UK government,[59] which could be regarded as an extraordinary lump-sum ex-ante premium. We also added the costs of a public tender for £3.3 million of ultra-low temperature freezers to the best-case scenario that was informed by the first authorised vaccine.[5] Although these costs could be considered as sunk costs and excluded from the analysis, we have included these costs given the counterfactual no-vaccination scenario to enable retrospective assessment of the value of these investments. Also, the impact of these cost factors on the overall cost-effectiveness results is negligible.

Additional cost factors could have been considered, including for instance an expanded testing programme or the running costs of the temporary field hospitals (estimated with approximately £15 million for the seven NHS Nightingale hospitals in England in April 2020).[60] While cost savings may be realisable on the field hospitals, expanded testing for SARS-CoV-2 may become a fixture in the years ahead for surveillance purposes irrespective of disease activity, and may thus be regarded as a fixed sunk-cost for economic analyses.

Supplementary Table 6: Input parameters in terms of costs			
Parameter	Value	Distribution	Sources
Costs per GP visit	£39.00	lognormal	Curtis and Burns (2019)[48]
Costs per remote helpline call (NHS111)	£13.86	lognormal	Turner et al. (2012);[50] inflated to 2018/19 using HS index[48]
Costs per non-ICU hospitalisation	<=18y: £1482.60; adults: £1770.38	lognormal	NHS reference costs (2018/19)[51]
Costs per ICU hospitalisation	<=18y: £2246.43; adults: £1504.47	lognormal	NHS reference costs (2018/19)[51]
Costs per PPE	£127.62	lognormal	PPE costs for MERS-CoV in 2015,[53] inflated to 2018/19 using HS index[48]
Costs per AEFI	£39.00	lognormal	assumed with costs of 1 GP visit per AEFI[48,55]
Costs per vaccine administration	£12.58	lognormal	service payment for COVID-19 (2020) [56]

Costs per vaccine dose	£15.00	fixed (varied in sensitivity analysis)	assumed cost-price similar to price reported for the first vaccine authorised for supply in the UK[5]
Costs for public tender of ultra-low temperature freezers	£3.3 m.	fixed, first year (i.e., undiscounted)	UK government reflecting the requirements of the first vaccine authorised for supply in the UK[5]
Costs of vaccination R&D (public funds)	£250.0 m.	fixed, first year (i.e., undiscounted)	UK government[59,61]
AEFI: adverse events following immunisation, COVID-19: coronavirus disease 2019, GP: general practitioner, HS: health services, ICU: intensive-care unit, MERS: Middle East respiratory syndrome, NHS: National Health Service, PPE: personal protective equipment, R&D: research and development, UK: United Kingdom of Great Britain and Northern Ireland			

Additional results

Supplementary Table 7: Results (mean, lower UI, upper UI) in terms of cases, deaths, and days spent in physical distancing per physical distancing (PD) scenario and vaccination scenario.

**Outcome**	**PD scenario**	**Vaccination scenario**		
		**A**	**B**	**C**
cases (in mln.)	1	147.99 (48.55, 198.83)	127.25 (28.70, 181.64)	37.93 (11.70, 112.95)
	2	141.89 (45.58, 193.53)	121.68 (25.17, 176.02)	33.94 (5.52, 106.36)
	3	21.38 (10.18, 98.30)	15.11 (9.79, 77.00)	12.11 (1.53, 26.00)
	4	30.55 (15.77, 98.90)	23.25 (21.06, 77.74)	14.08 (2.04, 33.90)
	5	37.36 (23.13, 99.19)	29.99 (22.74, 81.89)	16.94 (2.13, 38.04)
	6	43.53 (24.78, 103.50)	35.83 (22.75, 85.23)	18.63 (2.64, 44.04)
	7	48.68 (38.03, 106.07)	41.20 (22.84, 89.00)	20.24 (3.56, 50.24)
	8	52.82 (43.16, 108.72)	46.43 (22.94, 91.87)	21.39 (5.52, 56.67)
	9	70.15 (45.58, 118.35)	65.39 (25.17, 102.76)	23.42 (5.52, 87.86)
deaths (in mln.)	1	3.07 (0.84, 4.49)	2.55 (0.49, 3.97)	0.74 (0.21, 2.12)
	2	2.91 (0.78, 4.31)	2.40 (0.40, 3.78)	0.63 (0.08, 1.90)
	3	0.39 (0.17, 2.04)	0.27 (0.15, 1.53)	0.17 (0.02, 0.47)
	4	0.57 (0.26, 2.05)	0.42 (0.34, 1.55)	0.21 (0.03, 0.60)
	5	0.70 (0.38, 2.06)	0.54 (0.37, 1.64)	0.25 (0.03, 0.66)
	6	0.81 (0.41, 2.16)	0.65 (0.37, 1.71)	0.28 (0.04, 0.75)
	7	0.91 (0.64, 2.22)	0.75 (0.37, 1.80)	0.30 (0.05, 0.85)
	8	0.99 (0.73, 2.28)	0.84 (0.37, 1.86)	0.33 (0.08, 0.94)
	9	1.34 (0.78, 2.50)	1.21 (0.40, 2.10)	0.36 (0.08, 1.48)
days of increased PD	1	0.00 (0.00, 0.00)	0.00 (0.00, 0.00)	0.00 (0.00, 0.00)
	2	129.00 (49.00, 149.00)	129.00 (49.00, 149.00)	129.00 (49.00, 149.00)
	3	3381.00 (892.00, 3582.00)	3088.00 (435.00, 3582.00)	569.00 (89.00, 2341.00)
	4	3183.00 (778.00, 3583.00)	2905.00 (151.00, 3583.00)	527.00 (105.00, 2187.00)

	5	3039.00 (585.00, 3576.00)	2760.00 (105.00, 3467.00)	466.00 (103.00, 2062.00)
	6	2909.00 (549.00, 3458.00)	2633.00 (107.00, 3371.00)	434.00 (103.00, 1902.00)
	7	2803.00 (245.00, 3384.00)	2511.00 (107.00, 3263.00)	401.00 (100.00, 1732.00)
	8	2714.00 (111.00, 3307.00)	2390.00 (107.00, 3182.00)	379.00 (49.00, 1538.00)
	9	2331.00 (49.00, 3033.00)	1923.00 (49.00, 2876.00)	354.00 (49.00, 627.00)

Physical distancing scenarios: (1) no lockdown; (2) initial lockdown only; (3) PD trigger at 10/100,000 cases; (4) PD trigger at 20/100,000 cases; (5) PD trigger at 30/100,000 cases; (6) PD trigger at 40/100,000 cases; (7) PD trigger at 50/100,000 cases; (8) PD trigger at 60/100,000 cases; (9) PD trigger at 100/100,000 cases.

Vaccination scenarios: (A): no-vaccination baseline scenario; (B): vaccination with 50% vaccine effectiveness against disease, vaccine-induced protection of 45-weeks duration; (C): vaccination with 95% vaccine effectiveness against infection, vaccine-induced protection of 3-year duration.

mln.: million, PD: physical distancing, UI: uncertainty interval.

Supplementary Table 8: Results (mean, lower UI, upper UI) in terms of QALYs, costs, and net monetary value from the healthcare perspective per physical distancing (PD) and vaccination scenario.

**Outcome**	**PD scenario**	**Vaccination scenario**		
		**A**	**B**	**C**
QALYs (in bln.)	1	0.0931 (0.0775, 0.1102)	0.0789 (0.0655, 0.0932)	0.0258 (0.0215, 0.0304)
	2	0.0881 (0.0732, 0.1042)	0.0743 (0.0616, 0.0877)	0.0223 (0.0186, 0.0263)
	3	0.0124 (0.0103, 0.0147)	0.0093 (0.0078, 0.0109)	0.0068 (0.0057, 0.0080)
	4	0.0178 (0.0148, 0.0211)	0.0139 (0.0116, 0.0164)	0.0079 (0.0067, 0.0094)
	5	0.0218 (0.0181, 0.0258)	0.0178 (0.0149, 0.0209)	0.0096 (0.0080, 0.0113)
	6	0.0255 (0.0211, 0.0302)	0.0211 (0.0176, 0.0249)	0.0106 (0.0089, 0.0125)
	7	0.0285 (0.0236, 0.0337)	0.0242 (0.0201, 0.0286)	0.0115 (0.0097, 0.0137)
	8	0.0310 (0.0257, 0.0367)	0.0273 (0.0227, 0.0322)	0.0123 (0.0103, 0.0145)
	9	0.0415 (0.0344, 0.0491)	0.0383 (0.0318, 0.0451)	0.0136 (0.0114, 0.0161)
costs (in bln.)	1	85.62 (58.66, 123.82)	141.36 (115.92, 175.90)	92.44 (78.54, 112.02)
	2	80.95 (55.47, 117.04)	137.08 (113.12, 169.73)	89.26 (76.00, 107.38)
	3	11.36 (7.80, 16.40)	77.39 (65.05, 94.37)	75.01 (63.02, 92.19)
	4	16.29 (11.18, 23.51)	81.58 (68.88, 98.41)	76.04 (63.95, 92.99)
	5	20.00 (13.72, 28.86)	85.12 (72.26, 102.57)	77.54 (65.19, 94.51)
	6	23.35 (16.02, 33.70)	88.18 (74.90, 106.02)	78.44 (65.88, 95.43)

	7	26.12 (17.92, 37.70)	91.02 (77.45, 109.76)	79.32 (66.55, 96.23)
	8	28.41 (19.49, 41.01)	93.80 (79.61, 113.38)	79.99 (67.22, 96.84)
	9	37.99 (26.07, 54.86)	103.88 (87.84, 125.87)	81.23 (68.51, 98.12)
NMV (in bln.)	1	-487.12 (-632.02, -369.24)	-430.26 (-549.46, -327.90)	-152.41 (-189.17, -117.14)
	2	-460.55 (-597.39, -349.22)	-405.83 (-517.62, -309.50)	-134.21 (-165.45, -103.44)
	3	-64.92 (-84.06, -49.26)	-66.06 (-79.67, -51.68)	-52.90 (-63.60, -41.81)
	4	-93.05 (-120.50, -70.62)	-90.05 (-109.36, -69.93)	-58.86 (-71.00, -46.36)
	5	-114.18 (-147.88, -86.68)	-110.30 (-134.42, -85.33)	-67.48 (-81.71, -52.94)
	6	-133.30 (-172.65, -101.20)	-127.77 (-157.01, -98.60)	-72.69 (-88.18, -56.92)
	7	-149.11 (-193.14, -113.21)	-144.07 (-178.12, -110.99)	-77.76 (-94.45, -60.78)
	8	-162.17 (-210.06, -123.13)	-159.96 (-198.70, -123.07)	-81.61 (-99.22, -63.71)
	9	-216.96 (-281.11, -164.78)	-217.56 (-273.32, -166.83)	-88.70 (-107.99, -69.09)

Physical distancing scenarios: (1) no lockdown; (2) initial lockdown only; (3) PD trigger at 10/100,000 cases; (4) PD trigger at 20/100,000 cases; (5) PD trigger at 30/100,000 cases; (6) PD trigger at 40/100,000 cases; (7) PD trigger at 50/100,000 cases; (8) PD trigger at 60/100,000 cases; (9) PD trigger at 100/100,000 cases.

Vaccination scenarios: (A): no-vaccination baseline scenario; (B): vaccination with 50% vaccine effectiveness against disease, vaccine-induced protection of 45-weeks duration; (C): vaccination with 95% vaccine effectiveness against infection, vaccine-induced protection of 3-year duration.

bln.: billion, NMV: net monetary value, PD: physical distancing, QALY: quality-adjusted life year, UI: uncertainty interval.

Supplementary Table 9: Results in terms of total and incremental QALYs and costs, and the incremental cost-effectiveness ratio, from the healthcare perspective per physical distancing (PD) and vaccination scenario.

Results per PD scenario 1 to 9

	total costs, billion GBP (healthcare perspective)			incremental costs, billion GBP (healthcare persp.)			total QALYs, in billion			incremental QALYs, in billion			incremental cost-effectiveness ratios (ICERs)		
**PD scenario**	**A**	**B**	**C**	**B vs A**	**C vs A**	**C vs B**	**A**	**B**	**C**	**B vs A**	**C vs A**	**C vs B**	**B vs A**	**C vs A**	**C vs B**
1	85.62	141.36	92.44	55.74	6.82	-48.91	0.0931	0.0789	0.0258	0.0142	0.0673	0.0531	3,920	101	CS
2	80.95	137.08	89.26	56.13	8.31	-47.82	0.0881	0.0743	0.0223	0.0138	0.0657	0.0519	4,070	126	CS
3	11.36	77.39	75.01	66.03	63.65	-2.38	0.0124	0.0093	0.0068	0.0031	0.0056	0.0025	21,100	11,300	CS
4	16.29	81.58	76.04	65.29	59.75	-5.54	0.0178	0.0139	0.0079	0.0039	0.0099	0.0060	16,700	6,050	CS
5	20.00	85.12	77.54	65.13	57.54	-7.59	0.0218	0.0178	0.0096	0.0041	0.0123	0.0082	16,000	4,690	CS
6	23.35	88.18	78.44	64.83	55.09	-9.74	0.0255	0.0211	0.0106	0.0044	0.0149	0.0105	14,700	3,690	CS
7	26.12	91.02	79.32	64.91	53.20	-11.71	0.0285	0.0242	0.0115	0.0043	0.0170	0.0127	15,100	3,130	CS
8	28.41	93.80	79.99	65.40	51.59	-13.81	0.0310	0.0273	0.0123	0.0038	0.0187	0.0150	17,400	2,750	CS
9	37.99	103.88	81.23	65.89	43.24	-22.65	0.0415	0.0383	0.0136	0.0032	0.0279	0.0246	20,400	1,550	CS

Results per "no-lockdown" (PD scenario 1), no vaccination (scenario A)

	total costs, billion GBP (healthcare perspective)			incremental costs, billion GBP (healthcare persp.)			total QALYs, in billion			incremental QALYs, in billion			incremental cost-effectiveness ratios (ICERs)		
**PD scenario**	**A**	**B**	**C**	**A vs 1A**	**B vs 1A**	**C vs 1A**	**A**	**B**	**C**	**A vs 1A**	**B vs 1A**	**C vs 1A**	**A vs 1A**	**B vs 1A**	**C vs 1A**
1	85.62	141.36	92.44	Ref.	55.74	6.82	0.0931	0.0789	0.0258	Ref.	0.0142	0.0673	Ref.	3,920	101
2	80.95	137.08	89.26	-4.67	51.46	3.64	0.0881	0.0743	0.0223	0.0051	0.0189	0.0708	CS	2,730	51
3	11.36	77.39	75.01	-74.26	-8.23	-10.61	0.0124	0.0093	0.0068	0.0807	0.0839	0.0864	CS	CS	CS
4	16.29	81.58	76.04	-69.33	-4.04	-9.58	0.0178	0.0139	0.0079	0.0753	0.0793	0.0852	CS	CS	CS
5	20.00	85.12	77.54	-65.63	-0.50	-8.09	0.0218	0.0178	0.0096	0.0713	0.0754	0.0836	CS	CS	CS
	23.35	88.18	78.44	-62.28	2.55	-7.18	0.0255	0.0211	0.0106	0.0677	0.0720	0.0826	CS	35	CS
7	26.12	91.02	79.32	-59.51	5.40	-6.30	0.0285	0.0242	0.0115	0.0646	0.0689	0.0816	CS	78	CS
8	28.41	93.80	79.99	-57.22	8.18	-5.63	0.0310	0.0273	0.0123	0.0621	0.0659	0.0809	CS	124	CS
9	37.99	103.88	81.23	-47.63	18.26	-4.39	0.0415	0.0383	0.0136	0.0517	0.0549	0.0795	CS	333	CS

Physical distancing scenarios: (1) no lockdown; (2) initial lockdown only; (3) PD trigger at 10/100,000 cases; (4) PD trigger at 20/100,000 cases; (5) PD trigger at 30/100,000 cases; (6) PD trigger at 40/100,000 cases; (7) PD trigger at 50/100,000 cases; (8) PD trigger at 60/100,000 cases; (9) PD trigger at 100/100,000 cases.

Vaccination scenarios: (A): no-vaccination baseline scenario; (B): vaccination with 50% vaccine effectiveness against disease, vaccine-induced protection of 45-weeks duration; (C): vaccination with 95% vaccine effectiveness against infection, vaccine-induced protection of 3-year duration.

CS: cost-saving (fewer costs incurred at higher QALY losses prevented); GBP: British Pound Sterling, PD: physical distancing, QALY: quality-adjusted life year.

Sensitivity analyses

In a first sensitivity analysis, we explored an assumed vaccination effectiveness against disease and against infection according to the lower and upper bounds of the vaccine efficacy reported in the AstraZeneca-Oxford University phase 3 clinical trial (Supplementary Figure 1), and one according to the vaccine efficacy against disease of the Pfizer-BioNTech phase 3 clinical trial supplemented with reasonable assumptions for the vaccine effectiveness against infection (Supplementary Figure 2). Strategy B is similar to the base case analysis, while in strategy C transmission is ongoing over the ten years (Supplementary Figure 1-2).

In a second sensitivity analysis, we explored the epidemiological impact of vaccinating the targeted population uniformly. Compared to the targeted vaccination strategy using prioritisation groups (shown in the main text; Figure 1), vaccinating uniformly leads to similar disease dynamics in terms of epidemics but higher case numbers (Supplementary Figure 3).

In a third sensitivity analysis, we explored different uniform discount rates for benefits and costs (Supplementary Figure 4).

In a fourth sensitivity analysis, we explored the change in the net monetary value of vaccination if vaccine introduction is delayed up until the start of 2022. The economic value of introducing vaccination was estimated to be lower if the vaccine is introduced in December 2020 compared to an earlier introduction (Supplementary Figure 5). The incremental net monetary value of introducing vaccination does not always decrease as the delay increases, due to the interaction with seasonal cycles in incidence, holidays, and periods of physical distancing. In particular, vaccine introduction before an epidemic peak decreases the case numbers and height of said peak and leads to higher net values of vaccination, while vaccine introduction at the height of a peak or even afterwards will lead to lower net values of vaccination due to not having been able to prevent cases and the associated disease burden. This sensitivity analysis illustrates the (counterfactual) economic costs of delaying vaccination with a safe and effective vaccine, and assuming a vaccine has been authorised for supply by competent regulatory authorities with demonstrated effectiveness and safety from completed phase 3 trials.

In a fifth sensitivity analysis, we explored the incremental impact on the net monetary value in terms of efficiency per vaccinated individual in the targeted age groups using prioritisation groups or vaccinating uniformly (Supplementary Figure 6). The efficiency of a targeted programme in terms of incremental net monetary value per vaccinated individual can be increased by moving from older age groups to younger age groups (all of which start vaccinating the oldest ages first according to the JCVI prioritisation advice). For a uniform programme, the efficiency in terms of incremental net monetary value per vaccinated individual is largest when vaccinating the oldest age groups. These findings mostly align for the worst-case and best-case scenarios, although a disease-preventing vaccine may lead to negative incremental net values compared to no-vaccination.

In a sixth sensitivity analyses, we varied the re-vaccination coverage level in ages 15-64 years, with the net monetary value increasing with higher coverage levels for the best-case vaccination scenario but may become negative for the worst-case vaccination scenario (Supplementary Figure 7).

In a seventh sensitivity analysis, we further explored a different rate of vaccination uptake in the UK after the first prioritisation groups and before annual revaccinations. The results show that the speed of the initial vaccination rate has only a minor impact on results (Supplementary Figure 8).

In an eighth sensitivity analysis, we limited the importation rate of new infections to the first 6 months, which would in theory allow elimination. Results are not different from the base case as the locally transmitted cases rapidly exceed the low number of imported cases (Supplementary Figure 9).

Supplementary Figure 1: Epidemiological impact of vaccination effectiveness (AstraZeneca-Oxford University vaccine). Sensitivity analysis on the vaccination effectiveness according to the lower and upper bounds of the vaccine efficacy reported in the AstraZeneca-Oxford University phase 3 clinical trial. Days highlighted indicate summer and winter holidays (in light-blue), periods of physical distancing (light-red), or neither of the two (white). Note: The y-axis is truncated at 100,000 cases daily to allow meaningful visual comparisons across panels.

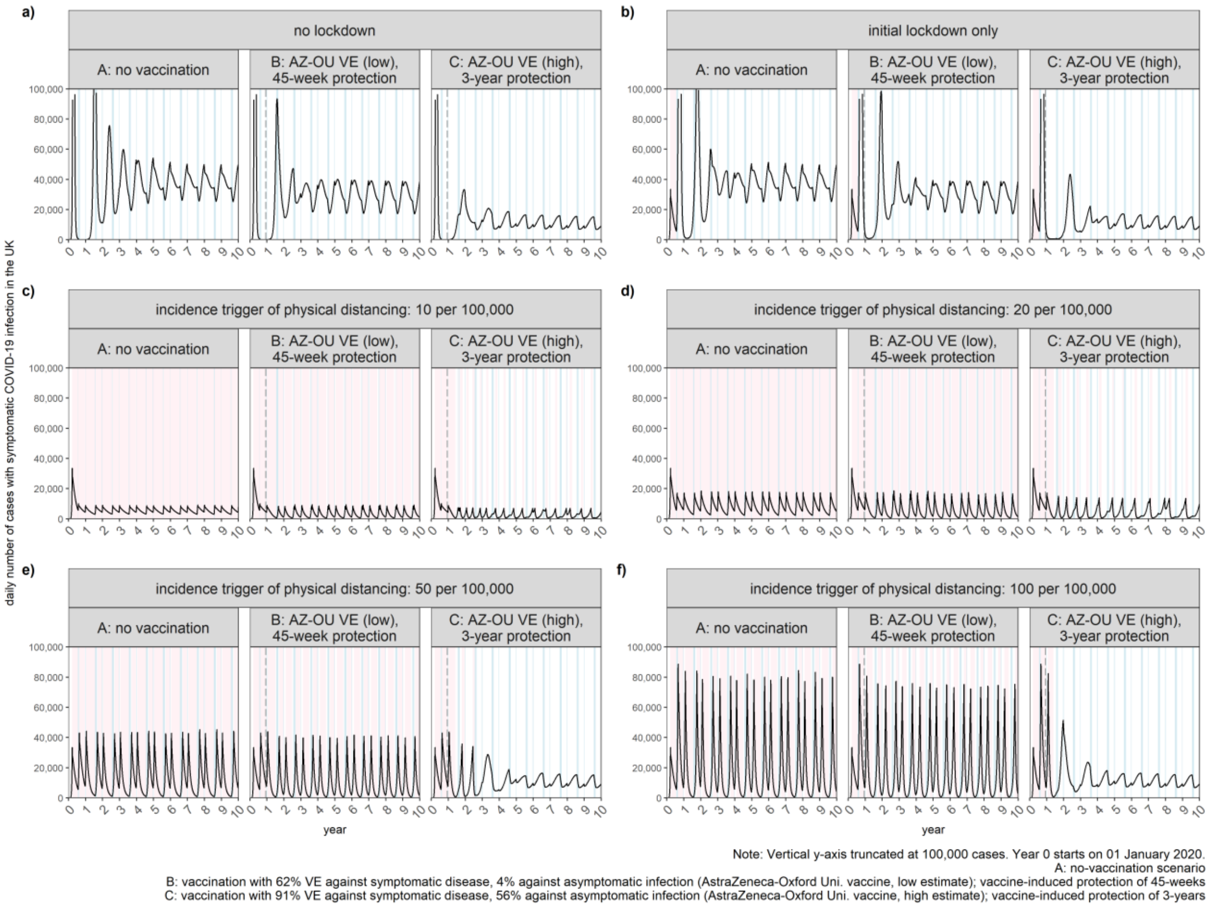

Supplementary Figure 2: Epidemiological impact of vaccination effectiveness (Pfizer-BioNTech vaccine). Sensitivity analysis on the vaccination effectiveness according to the vaccine efficacy against disease of the Pfizer-BioNTech phase 3 clinical trial supplemented with reasonable assumptions for the vaccine effectiveness against infection. Days highlighted indicate summer and winter holidays (in light-blue), periods of physical distancing (light-red), or neither of the two (white). Note: The y-axis is truncated at 100,000 cases daily to allow meaningful visual comparisons across panels.

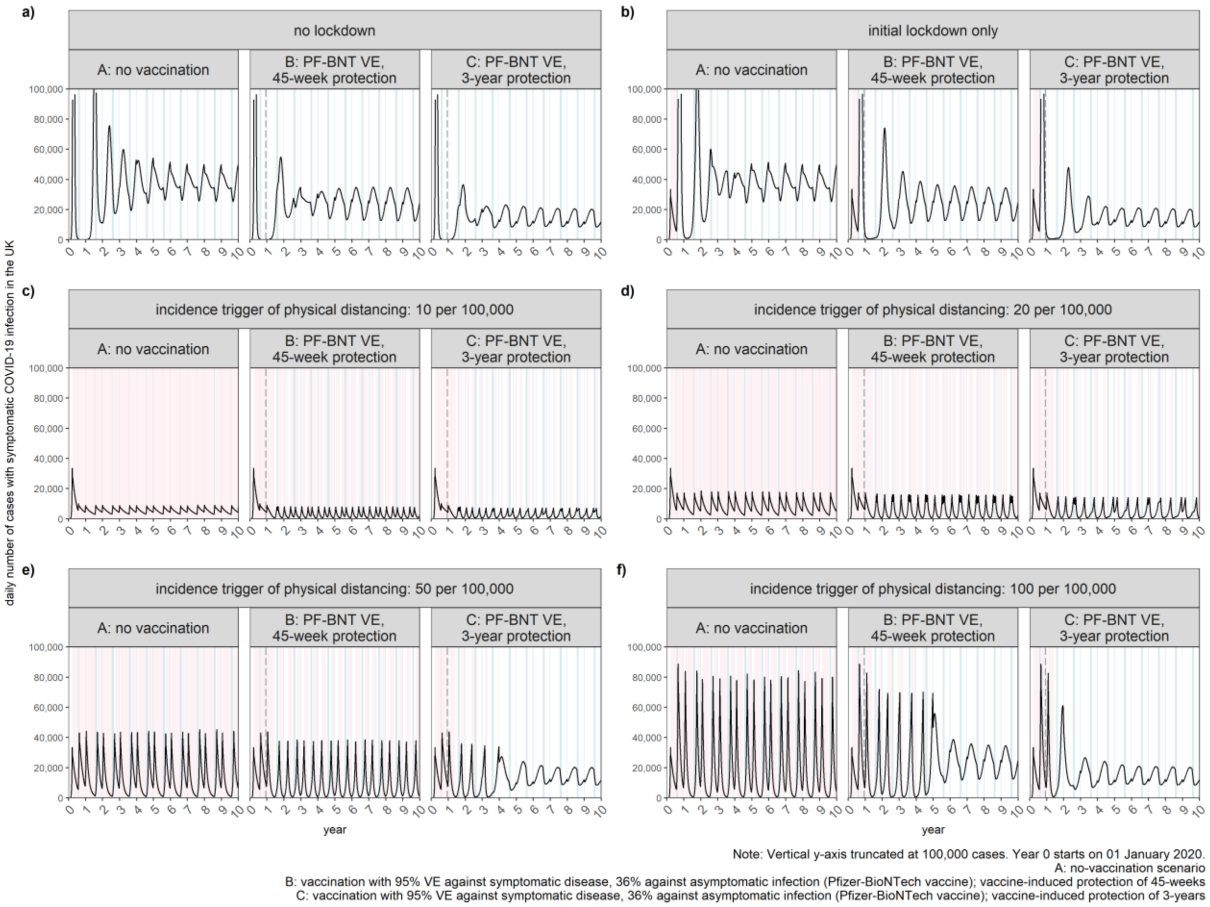

Supplementary Figure 3: Vaccinating uniformly. Sensitivity analysis on the vaccination programme when vaccinating uniformly (instead of targeted vaccination using prioritisation groups as shown in the main text). Days highlighted indicate summer and winter holidays (in light-blue), periods of physical distancing (light-red), or neither of the two (white). Note: The y-axis is truncated at 100,000 cases daily to allow meaningful visual comparisons across panels.

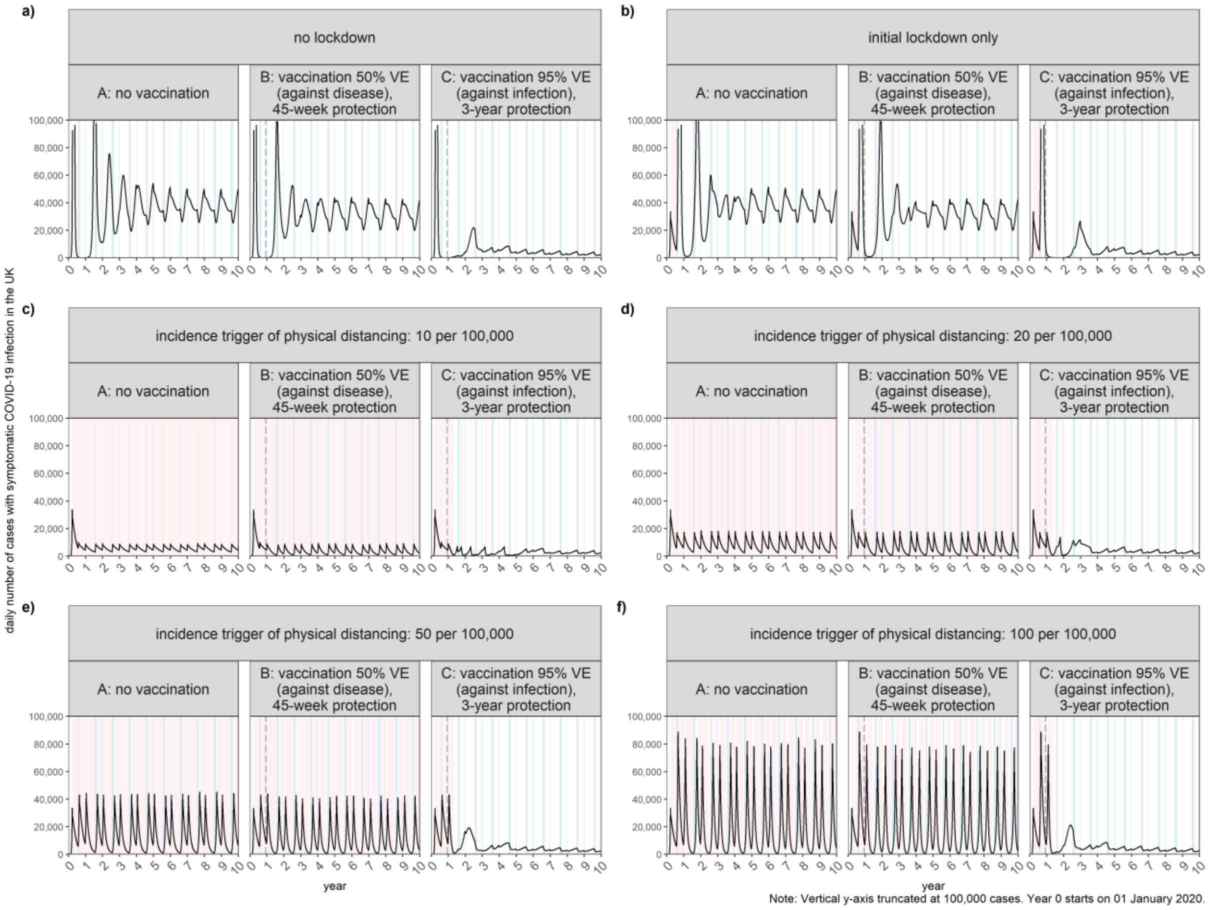

Supplementary Figure 4: Sensitivity analysis on the discount rate.

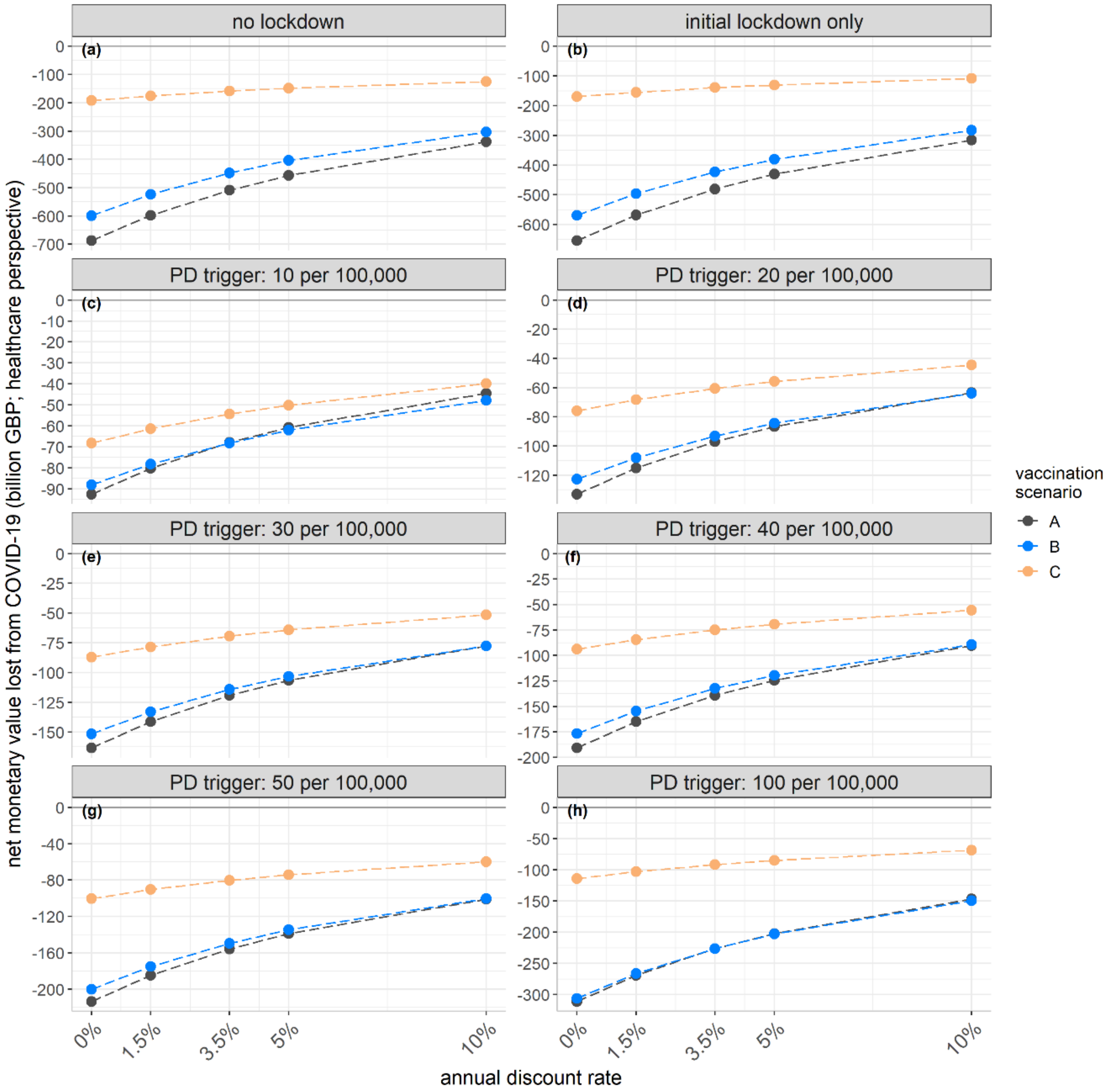

Net monetary values (QALYs * £20,000 - costs; discounted at 3.5% over 10 years) are negative due to health losses and costs.
A: no-vaccination scenario
B: vaccination with 50% VE against disease, vaccine-induced protection of 45-weeks
C: vaccination with 95% VE against infection, vaccine-induced protection of 3-years
Natural protection of 45-weeks, uptake of 75% (15+ y.), coverage of 50% (15-64 y.) and 75% (65+ y.)

Supplementary Figure 5: Timing of vaccination. Sensitivity analysis on the timing of vaccination introduction in terms of changes to the net monetary value when introducing vaccination in December 2020.

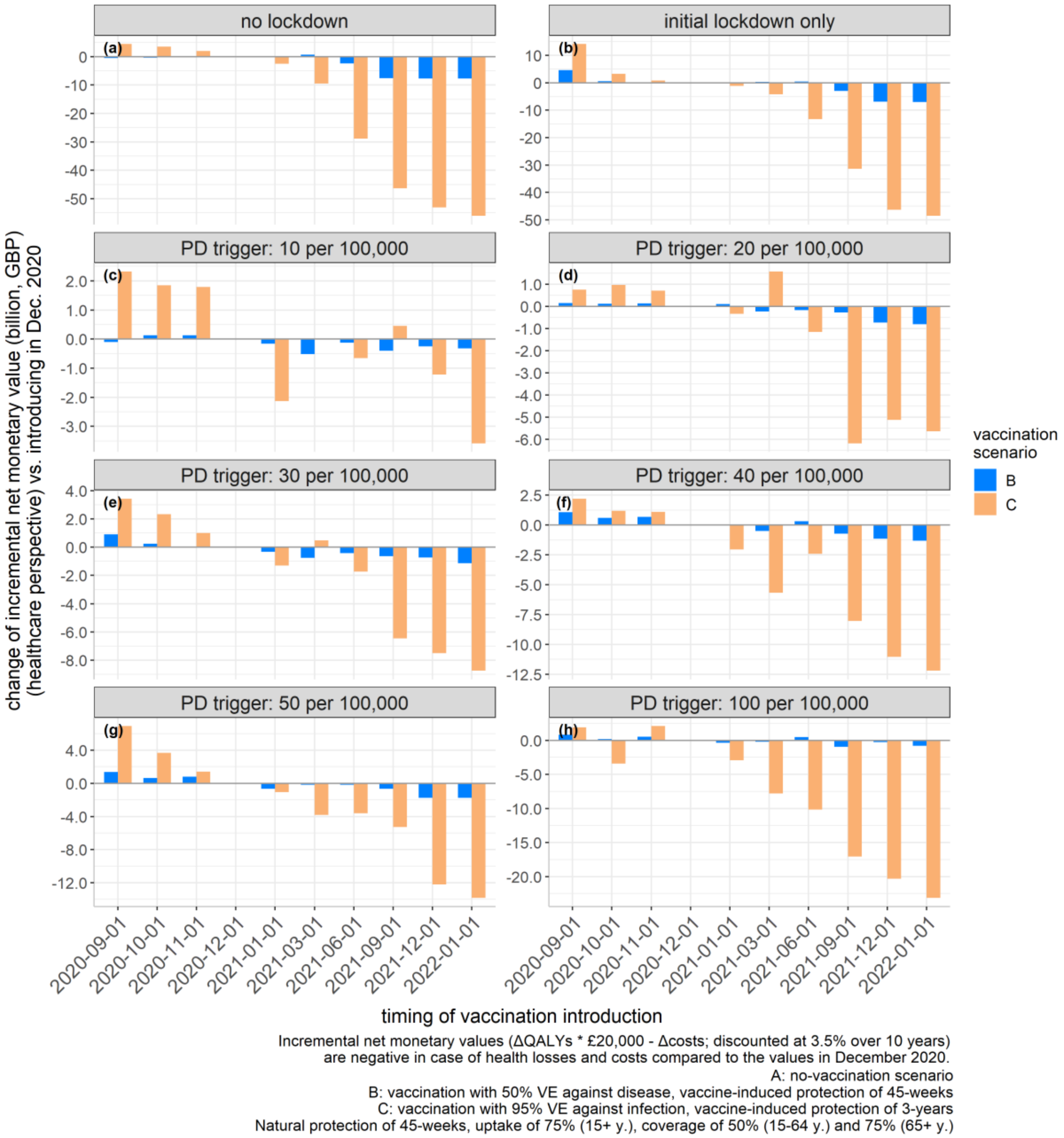

Supplementary Figure 6: Age group of vaccination and target groups. Sensitivity analysis on the age groups targeted in the vaccination programme scenarios B and C versus the no-vaccination scenario A using prioritisation groups (a-f) or vaccinating uniformly (g-l).

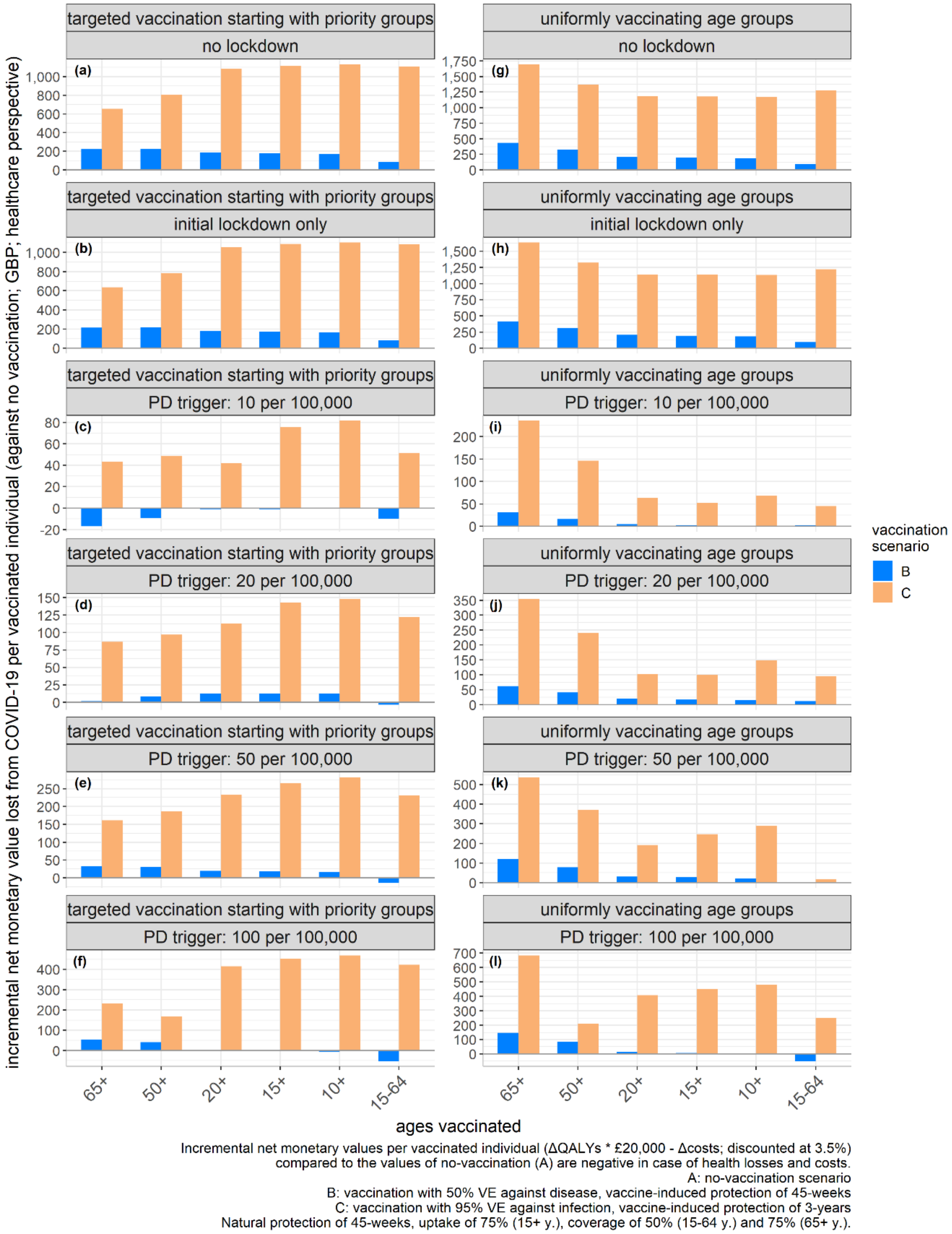

Supplementary Figure 7: Re-vaccination coverage level. Sensitivity analysis on the re-vaccination coverage level.

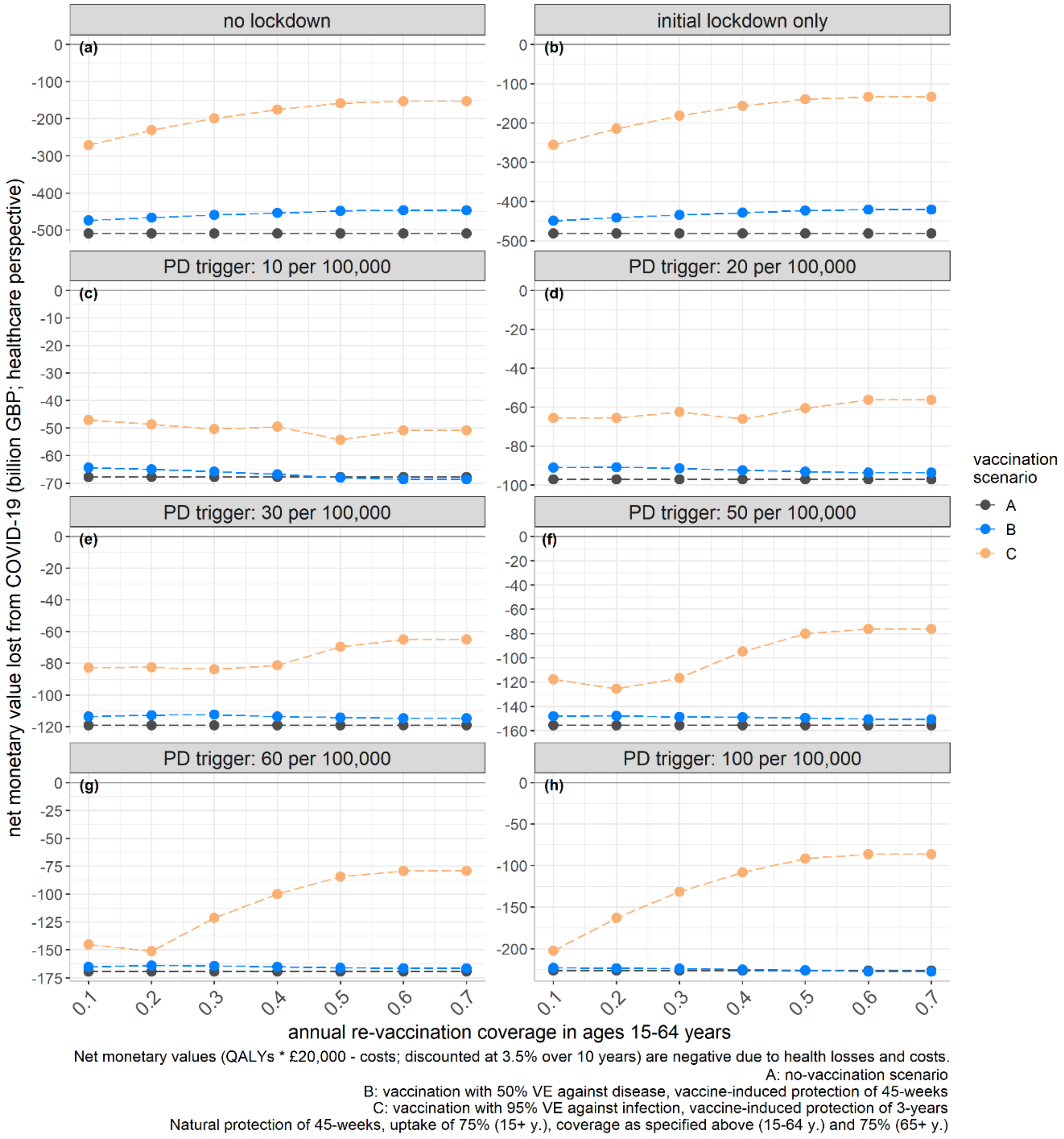

Supplementary Figure 8: Vaccination uptake rate. Sensitivity analysis on the vaccination uptake rate after the initial phase and before starting annual re-vaccinations.

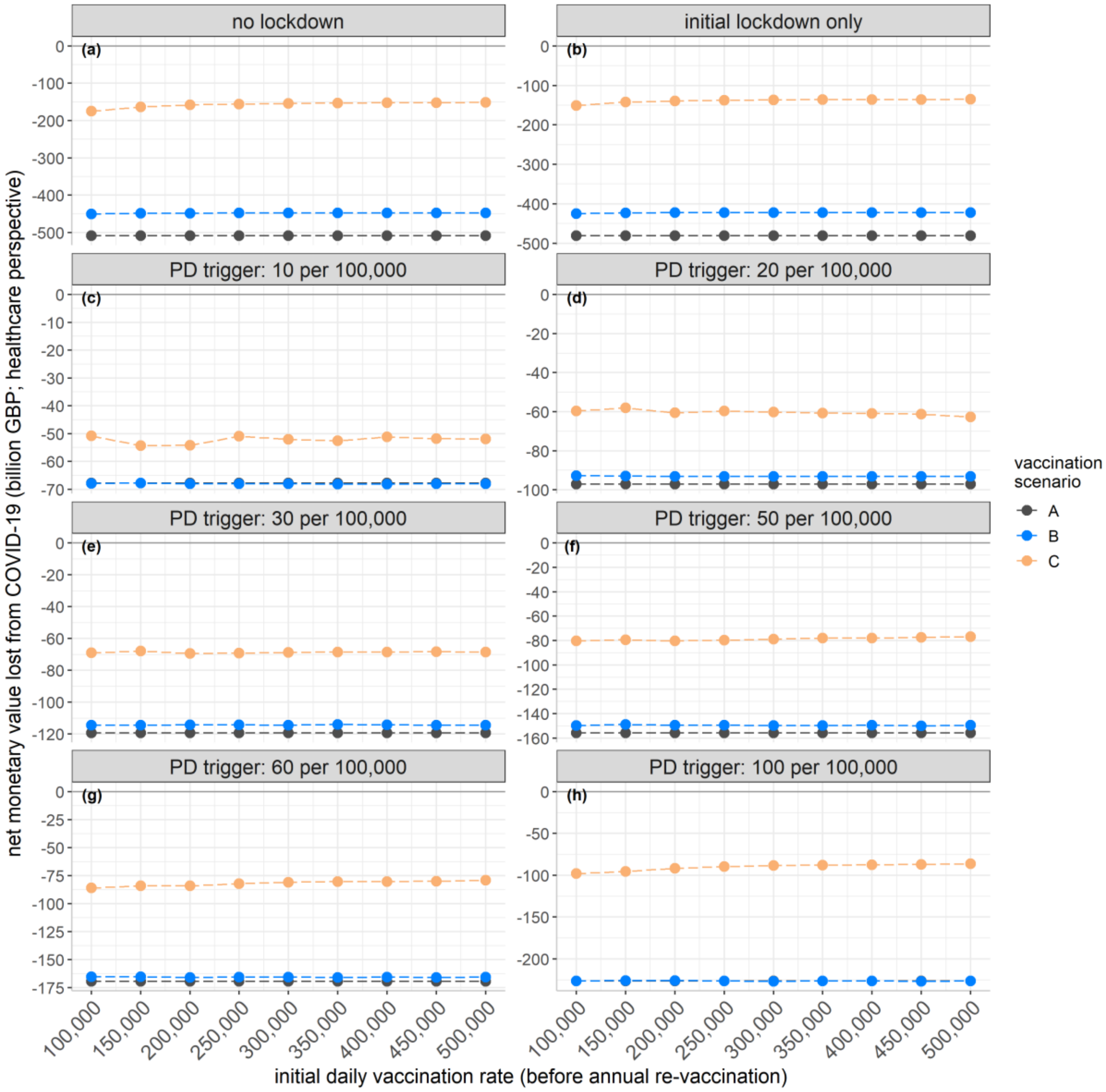

Net monetary values (QALYs * £20,000 - costs; discounted at 3.5% over 10 years) are negative due to health losses and costs.
A: no-vaccination scenario
B: vaccination with 50% VE against disease, vaccine-induced protection of 45-weeks
C: vaccination with 95% VE against infection, vaccine-induced protection of 3-years
Natural protection of 45-weeks, uptake of 75% (15+ y.), coverage of 50% (15-64 y.) and 75% (65+ y.).

Supplementary Figure 9: Epidemiological impact of imported cases. Sensitivity analysis on the number of imported cases each month (limited to the initial six months). Days highlighted indicate summer and winter holidays (in light-blue), periods of physical distancing (light-red), or neither of the two (white). Note: The y-axis is truncated at 100,000 cases daily to allow meaningful visual comparisons across panels.

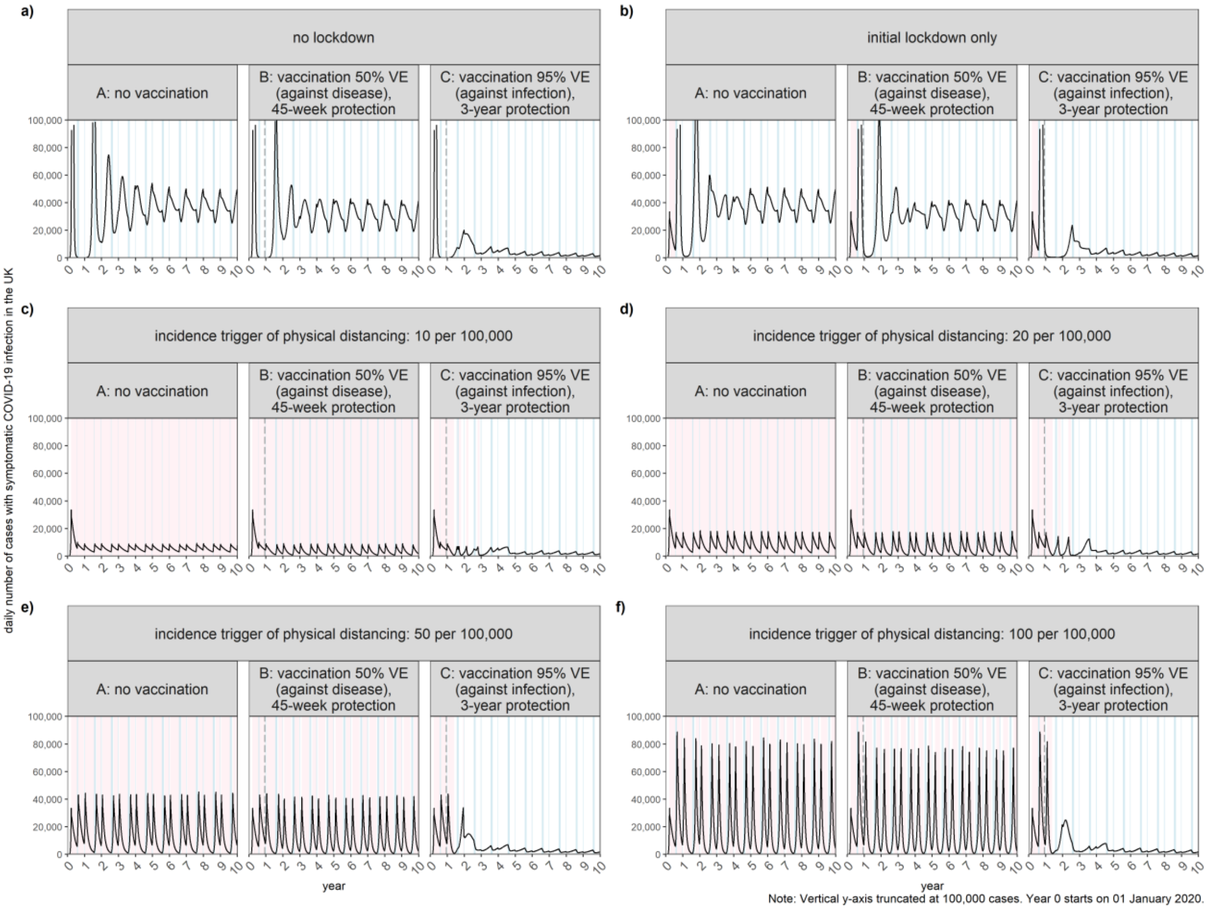

CHEERS checklist - Items to include when reporting economic evaluations of health interventions

Section/item	Item No	Recommendation	Reported on page No/ line No
**Title and abstract**			
Title	1	Identify the study as an economic evaluation or use more specific terms such as "cost-effectiveness analysis", and describe the interventions compared.	Title, p. 1
Abstract	2	Provide a structured summary of objectives, perspective, setting, methods (including study design and inputs), results (including base case and uncertainty analyses), and conclusions.	Abstract, p. 1
**Introduction**			
Background and objectives	3	Provide an explicit statement of the broader context for the study.	Introduction, p. 3
		Present the study question and its relevance for health policy or practice decisions.	Introduction, p. 3
**Methods**			
Target population and subgroups	4	Describe characteristics of the base case population and subgroups analysed, including why they were chosen.	Methods, p. 4
Setting and location	5	State relevant aspects of the system(s) in which the decision(s) need(s) to be made.	Methods, p. 4
Study perspective	6	Describe the perspective of the study and relate this to the costs being evaluated.	Methods, pp. 5-6
Comparators	7	Describe the interventions or strategies being compared and state why they were chosen.	Methods, pp. 4-5
Time horizon	8	State the time horizon(s) over which costs and consequences are being evaluated and say why appropriate.	Methods, p. 5
Discount rate	9	Report the choice of discount rate(s) used for costs and outcomes and say why appropriate.	Methods, p. 5
Choice of health outcomes	10	Describe what outcomes were used as the measure(s) of benefit in the evaluation and their relevance for the type of analysis performed.	Methods, pp. 5-6
Measurement of effectiveness	11a	*Single study-based estimates:* Describe fully the design features of the single effectiveness study and why the single study was a sufficient source of clinical effectiveness data.	n/a
	11b	*Synthesis-based estimates:* Describe fully the methods used for identification of included studies and synthesis of clinical effectiveness data.	Methods, pp. 4; appendix pp. 1-7
Measurement and valuation of preference based outcomes	12	If applicable, describe the population and methods used to elicit preferences for outcomes.	Methods, pp. 5-6; appendix pp. 8-9

Section/item	Item No	Recommendation	Reported on page No/ line No
Estimating resources and costs	13a	*Single study-based economic evaluation:* Describe approaches used to estimate resource use associated with the alternative interventions. Describe primary or secondary research methods for valuing each resource item in terms of its unit cost. Describe any adjustments made to approximate to opportunity costs.	n/a
	13b	*Model-based economic evaluation:* Describe approaches and data sources used to estimate resource use associated with model health states. Describe primary or secondary research methods for valuing each resource item in terms of its unit cost. Describe any adjustments made to approximate to opportunity costs.	Methods, pp. 5-6; appendix pp. 9-11
Currency, price date, and conversion	14	Report the dates of the estimated resource quantities and unit costs. Describe methods for adjusting estimated unit costs to the year of reported costs if necessary. Describe methods for converting costs into a common currency base and the exchange rate.	appendix pp. 9-11
Choice of model	15	Describe and give reasons for the specific type of decision-analytical model used. Providing a figure to show model structure is strongly recommended.	Methods, pp. 4-5; appendix pp. 1-5
Assumptions	16	Describe all structural or other assumptions underpinning the decision-analytical model.	Methods, pp. 4-5; appendix pp. 1-5
Analytical methods	17	Describe all analytical methods supporting the evaluation. This could include methods for dealing with skewed, missing, or censored data; extrapolation methods; methods for pooling data; approaches to validate or make adjustments (such as half cycle corrections) to a model; and methods for handling population heterogeneity and uncertainty.	Methods, pp. 4-6; appendix pp. 1-11
**Results**			
Study parameters	18	Report the values, ranges, references, and, if used, probability distributions for all parameters. Report reasons or sources for distributions used to represent uncertainty where appropriate. Providing a table to show the input values is strongly recommended.	Methods, pp. 4-6; appendix pp. 1-11
Incremental costs and outcomes	19	For each intervention, report mean values for the main categories of estimated costs and outcomes of interest, as well as mean differences between the comparator groups. If applicable, report incremental cost-effectiveness ratios.	Results, pp. 7-8; appendix pp. 11-14
Characterising uncertainty	20a	*Single study-based economic evaluation:* Describe the effects of sampling uncertainty for the estimated incremental cost and incremental effectiveness parameters, together with the impact of methodological assumptions (such as discount rate, study perspective).	n/a

Section/item	Item No	Recommendation	Reported on page No/ line No
	20b	*Model-based economic evaluation:* Describe the effects on the results of uncertainty for all input parameters, and uncertainty related to the structure of the model and assumptions.	Results, pp. 7-15; appendix pp. 15-24
Characterising heterogeneity	21	If applicable, report differences in costs, outcomes, or cost-effectiveness that can be explained by variations between subgroups of patients with different baseline characteristics or other observed variability in effects that are not reducible by more information.	Results, pp. 7-15; appendix pp. 15-24
**Discussion**			
Study findings, limitations, generalisability, and current knowledge	22	Summarise key study findings and describe how they support the conclusions reached. Discuss limitations and the generalisability of the findings and how the findings fit with current knowledge.	Discussion, p. 16-17
**Other**			
Source of funding	23	Describe how the study was funded and the role of the funder in the identification, design, conduct, and reporting of the analysis. Describe other non-monetary sources of support.	Methods, p. 6
Conflicts of interest	24	Describe any potential for conflict of interest of study contributors in accordance with journal policy. In the absence of a journal policy, we recommend authors comply with International Committee of Medical Journal Editors recommendations.	Title page, p. 1

For consistency, the CHEERS statement checklist format is based on the format of the CONSORT statement checklist

References

1. Public Health England and NHSX. Coronavirus (COVID-19) in the UK. 2021. https://coronavirus.data.gov.uk/ (accessed 25.01.2021.
2. Mossong J, Hens N, Jit M, et al. Social Contacts and Mixing Patterns Relevant to the Spread of Infectious Diseases. *PLOS Medicine* 2008; **5**(3): e74.
3. Jarvis CI, Van Zandvoort K, Gimma A, et al. Quantifying the impact of physical distance measures on the transmission of COVID-19 in the UK. *BMC Medicine* 2020; **18**(1): 124.
4. Public Health England (PHE). COVID-19: the green book, chapter 14a. 21.01.2021 2021. https://assets.publishing.service.gov.uk/government/uploads/system/uploads/attachment_data/file/954724/Greenbook_chapter_14a_v5.pdf (accessed 25.01.2021.
5. Mahase E. Covid-19: What do we know about the late stage vaccine candidates? *BMJ* 2020; **371**: m4576.
6. Medicines and Healthcare products Regulatory Agency (MHRA). Regulaton 174 - Information for Healthcare Professionals on Pfizer/BioNTech COVID-19 Vaccine. 2020. https://assets.publishing.service.gov.uk/government/uploads/system/uploads/attachment_data/file/940565/Information_for_Healthcare_Professionals_on_Pfizer_BioNTech_COVID-19_vaccine.pdf (accessed 06.12.2020.
7. US Food and Drug Administration (FDA). Vaccines and Related Biological Products Advisory Committee Meeting; Pfizer-BioNTech COVID-19 Vaccine. 2020.
8. Polack FP, Thomas SJ, Kitchin N, et al. Safety and Efficacy of the BNT162b2 mRNA Covid-19 Vaccine. *NEJM* 2020.
9. (FDA) UFaDA. Vaccines and Related Biological Products Advisory Committee Meeting, December 17, 2020; FDA Briefing Document: Moderna COVID-19 Vaccine. 2020.
10. Merryn Voysey* SACC, Shabir A Madhi*, Lily Y Weckx*, Pedro M Folegatti*, Parvinder K Aley, Brian Angus, Vicky L Baillie,, Shaun L Barnabas QEB, Sagida Bibi, Carmen Briner, Paola Cicconi, Andrea M Collins, Rachel Colin-Jones, Clare L Cutland,, Thomas C Darton KD, Christopher J A Duncan, Katherine R W Emary, Katie J Ewer, Lee Fairlie, Saul N Faust, Shuo Feng,, et al. Safety and efficacy of the ChAdOx1 nCoV-19 vaccine (AZD1222) against SARS-CoV-2: an interim analysis of four randomised controlled trials in Brazil, South Africa, and the UK. *Lancet* 2020.
11. Medicines and Healthcare products Regulatory Agency (MHRA). Information for Healthcare Professionals on COVID-19 Vaccine Moderna. 2021. https://www.gov.uk/government/publications/regulatory-approval-of-covid-19-vaccine-moderna/information-for-healthcare-professionals-on-covid-19-vaccine-moderna (accessed 25.01.2021.
12. van Doremalen N, Lambe T, Spencer A, et al. ChAdOx1 nCoV-19 vaccine prevents SARS-CoV-2 pneumonia in rhesus macaques. *Nature* 2020; **586**(7830): 578-82.
13. Mercado NB, Zahn R, Wegmann F, et al. Single-shot Ad26 vaccine protects against SARS-CoV-2 in rhesus macaques. *Nature* 2020; **586**(7830): 583-8.
14. Krause P, Fleming TR, Longini I, et al. COVID-19 vaccine trials should seek worthwhile efficacy. *The Lancet* 2020; **396**(10253): 741-3.
15. Belongia EA, Simpson MD, King JP, et al. Variable influenza vaccine effectiveness by subtype: a systematic review and meta-analysis of test-negative design studies. *The Lancet Infectious diseases* 2016; **16**(8): 942-51.
16. Ward H, Cooke G, Atchison C, et al. Declining prevalence of antibody positivity to SARS-CoV-2: a community study of 365,000 adults. *medRxiv* 2020: 2020.10.26.20219725.
17. Dan JM, Mateus J, Kato Y, et al. Immunological memory to SARS-CoV-2 assessed for up to 8 months after infection. *Science* 2021: eabf4063.
18. Kissler SM, Tedijanto C, Goldstein E, Grad YH, Lipsitch M. Projecting the transmission dynamics of SARS-CoV-2 through the postpandemic period. *Science* 2020; **368**(6493): 860-8.
19. Hall V, Foulkes S, Charlett A, et al. Do antibody positive healthcare workers have lower SARS-CoV-2 infection rates than antibody negative healthcare workers? Large multi-centre prospective cohort study (the SIREN study), England: June to November 2020. *medRxiv* 2021: 2021.01.13.21249642.
20. Public Health England (PHE). Joint Committee on Vaccination and Immunisation: advice on priority groups for COVID-19 vaccination - 2 December 2020. 2020.

https://assets.publishing.service.gov.uk/government/uploads/system/uploads/attachment_data/file/940396/Priority_groups_for_coronavirus__COVID-19__vaccination_-_advice_from_the_JCVI__2_December_2020.pdf (accessed 02.12.2020.
21. Office for National Statistics (ONS). Changes in the Older Resident Care Home Population between 2001 and 2011. 2014. https://www.ons.gov.uk/peoplepopulationandcommunity/birthsdeathsandmarriages/ageing/articles/changesintheolderresidentcarehomepopulationbetween2001and2011/2014-08-01 (accessed 28.11.2020.
22. Skills for Care Ltd. The state of the adult social care sector and workforce in England. 2020. https://www.skillsforcare.org.uk/adult-social-care-workforce-data/Workforce-intelligence/publications/national-information/The-state-of-the-adult-social-care-sector-and-workforce-in-England.aspx (accessed 28.11.2020.
23. NHS Digital. NHS Workforce Statistics - August 2020. 2020. https://digital.nhs.uk/data-and-information/publications/statistical/nhs-workforce-statistics/august-2020 (accessed 28.11.2020.
24. Clark A, Jit M, Warren-Gash C, et al. Global, regional, and national estimates of the population at increased risk of severe COVID-19 due to underlying health conditions in 2020: a modelling study. *The Lancet Global Health* 2020; **8**(8): e1003-e17.
25. Sherman SM, Smith LE, Sim J, et al. COVID-19 vaccination intention in the UK: results from the COVID-19 vaccination acceptability study (CoVAccS), a nationally representative cross-sectional survey. *Human Vaccines & Immunotherapeutics* 2020: 1-10.
26. British Broadcasting Corporation (BBC). Covid-19 vaccine: First person receives Pfizer jab in UK. 2020. https://www.bbc.co.uk/news/uk-55227325 (accessed 08.12.2020.
27. NHS England. Missed GP appointments costing NHS millions. 2019. https://www.england.nhs.uk/2019/01/missed-gp-appointments-costing-nhs-millions/.
28. Public Health England (PHE). COVID-19 vaccination programme: Information for healthcare practitioners. 2020. https://assets.publishing.service.gov.uk/government/uploads/system/uploads/attachment_data/file/941236/COVID-19_vaccination_programme_guidance_for_healthcare_workers_December_2020_V2.pdf (accessed 04.12.2020.
29. Davies NG, Kucharski AJ, Eggo RM, et al. Effects of non-pharmaceutical interventions on COVID-19 cases, deaths, and demand for hospital services in the UK: a modelling study. *The Lancet Public Health* 2020; **5**(7): e375-e85.
30. Office for National Statistics (ONS). Overview of the UK population: January 2021. 2021. https://www.ons.gov.uk/peoplepopulationandcommunity/populationandmigration/populationestimates/articles/overviewoftheukpopulation/january2021 (accessed 25.01.2021.
31. Docherty AB, Harrison EM, Green CA, et al. Features of 20 133 UK patients in hospital with covid-19 using the ISARIC WHO Clinical Characterisation Protocol: prospective observational cohort study. 2020; **369**: m1985.
32. Davies NG, Klepac P, Liu Y, et al. Age-dependent effects in the transmission and control of COVID-19 epidemics. *Nature Medicine* 2020; **26**(8): 1205-11.
33. O'Driscoll M, Dos Santos GR, Wang L, et al. Age-specific mortality and immunity patterns of SARS-CoV-2. *Nature* 2020.
34. Ward H, Atchison CJ, Whitaker M, et al. Antibody prevalence for SARS-CoV-2 in England following first peak of the pandemic: REACT2 study in 100,000 adults. *medRxiv* 2020: 2020.08.12.20173690.
35. Carfì A, Bernabei R, Landi F, Group ftGAC-P-ACS. Persistent Symptoms in Patients After Acute COVID-19. *JAMA* 2020.
36. van Hoek AJ, Underwood A, Jit M, Miller E, Edmunds WJ. The impact of pandemic influenza H1N1 on health-related quality of life: a prospective population-based study. *PLoS One* 2011; **6**(3): e17030-e.
37. Halpin SJ, McIvor C, Whyatt G, et al. Postdischarge symptoms and rehabilitation needs in survivors of COVID-19 infection: A cross-sectional evaluation. **n/a**(n/a).
38. Baguelin M, Camacho A, Flasche S, Edmunds WJ. Extending the elderly- and risk-group programme of vaccination against seasonal influenza in England and Wales: a cost-effectiveness study. *BMC Med* 2015; **13**: 236.

39. Griffiths J, Hatch RA, Bishop J, et al. An exploration of social and economic outcome and associated health-related quality of life after critical illness in general intensive care unit survivors: a 12-month follow-up study. *Critical Care* 2013; **17**(3): R100.
40. Cuthbertson BH, Roughton S, Jenkinson D, MacLennan G, Vale L. Quality of life in the five years after intensive care: a cohort study. *Critical Care* 2010; **14**(1): R6.
41. Wyper GM, Assunção RM, Colzani E, et al. Burden of disease methods: a guide to calculate COVID-19 disability-adjusted life years. 2020.
42. Greenhalgh T, Knight M, A'Court C, Buxton M, Husain L. Management of post-acute covid-19 in primary care. 2020; **370**: m3026.
43. Raviotta JM, Smith KJ, DePasse J, et al. Cost-Effectiveness and Public Health Effect of Influenza Vaccine Strategies for U.S. Elderly Adults. *J Am Geriatr Soc* 2016; **64**(10): 2126-31.
44. Office for National Statistics (ONS). National life tables: United Kingdom. 2020. https://www.ons.gov.uk/peoplepopulationandcommunity/birthsdeathsandmarriages/lifeexpectancies/datasets/nationallifetablesunitedkingdomreferencetables (accessed 25.01.2021.
45. Ara R, Brazier JE. Populating an economic model with health state utility values: moving toward better practice. *Value Health* 2010; **13**(5): 509-18.
46. Briggs A. Moving beyond 'lives-saved' from COVID-19. 2020. https://www.lshtm.ac.uk/research/centres-projects-groups/chil#covid-19 (accessed 28/07/2020.
47. Briggs AH, Goldstein DA, Kirwin E, et al. Estimating (quality-adjusted) life-year losses associated with deaths: With application to COVID-19. 2020.
48. Curtis L, Burns A. Unit Costs of Health and Social Care 2019. Canterbury: Personal Social Services Research Unit, University of Kent; 2019.
49. Public Health England (PHE). National COVID-19 surveillance reports. 2020. https://www.gov.uk/government/publications/national-covid-19-surveillance-reports (accessed 11/08/2020.
50. Turner J, O'Cathain A, Knowles E, et al. Evaluation of NHS 111 pilot sites. Final Report to the Department of Health. 2012.
51. NHS Improvement. National Cost Collection for the NHS. 2020. https://improvement.nhs.uk/resources/national-cost-collection/#ncc1819 (accessed 11/08/2020.
52. Lau K, Hauck K, Miraldo M. Excess influenza hospital admissions and costs due to the 2009 H1N1 pandemic in England. *Health economics* 2019; **28**(2): 175-88.
53. Veater J, Wong N, Stephenson I, et al. Resource impact of managing suspected Middle East respiratory syndrome patients in a UK teaching hospital. *Journal of Hospital Infection* 2017; **95**(3): 280-5.
54. National Institute for Health and Care Excellence (NICE). Safe staffing for nursing in adult inpatient wards in acute hospitals. London, UK: National Institute for Health and Care Excellence (NICE), 2014.
55. Prosser LA, Bridges CB, Uyeki TM, et al. Health benefits, risks, and cost-effectiveness of influenza vaccination of children. *Emerg Infect Dis* 2006; **12**(10): 1548-58.
56. National Health Service (NHS) England. Enhanced Service Specification: COVID-19 vaccination programme 2020/21. 2020. https://www.england.nhs.uk/coronavirus/publication/ess-vaccination-programme/ (accessed 08.12.2020.
57. National Health Service (NHS) England. Enhanced service specifications. 2019. https://www.england.nhs.uk/publication/enhanced-service-specifications/ (accessed 02/04/2019.
58. British Medical Association (BMA). COVID-19 vaccination programme. 2021. https://www.bma.org.uk/advice-and-support/covid-19/vaccines/covid-19-vaccination-programme.
59. Department for Business EIS. Funding and manufacturing boost for UK vaccine programme. 2020. https://www.gov.uk/government/news/funding-and-manufacturing-boost-for-uk-vaccine-programme (accessed 05/08/2020.
60. Department of Health and Social Care (DHSC). Freedom of Information Request Reference FOI-1223558. 08/06/2020 2020. https://www.whatdotheyknow.com/request/663081/response/1583624/attach/3/FOI%201223558.pdf (accessed 03/08/2020.

61. Department for Business EIS. UK government secures new COVID-19 vaccines and backs global clinical trial. 2020. https://www.gov.uk/government/news/uk-government-secures-new-covid-19-vaccines-and-backs-global-clinical-trial (accessed 20.08.2020.

Centre for Mathematical Modelling of Infectious Disease COVID-19 working group

The following authors were part of the Centre for Mathematical Modelling of Infectious Disease COVID-19 Working Group. Each contributed in processing, cleaning and interpretation of data, interpreted findings, contributed to the manuscript, and approved the work for publication:

Fiona Yueqian Sun, C Julian Villabona-Arenas, Emily S Nightingale, Alicia Showering, Gwenan M Knight, Katharine Sherratt, Yang Liu, Kaja Abbas, Sebastian Funk, Akira Endo, Joel Hellewell, Alicia Rosello, Rachel Lowe, Matthew Quaife, Amy Gimma, Oliver Brady, Jack Williams, Simon R Procter, Rosalind M Eggo, Yung-Wai Desmond Chan, James D Munday, Rosanna C Barnard, Georgia R Gore-Langton, Nikos I Bosse, Naomi R Waterlow, Charlie Diamond, Timothy W Russell, Graham Medley, Stefan Flasche, Katherine E. Atkins, Kiesha Prem, David Simons, Megan Auzenbergs, Damien C Tully, Christopher I Jarvis, Kevin van Zandvoort, Sam Abbott, Carl A B Pearson, Thibaut Jombart, Sophie R Meakin, Anna M Foss, Adam J Kucharski, Billy J Quilty, Hamish P Gibbs, Samuel Clifford, Petra Klepac.
